# Structural Identification of Antibacterial Lipids from Amazonian Palm Tree Endophytes through the Molecular Network Approach

**DOI:** 10.3390/ijms20082006

**Published:** 2019-04-24

**Authors:** Morgane Barthélemy, Nicolas Elie, Léonie Pellissier, Jean-Luc Wolfender, Didier Stien, David Touboul, Véronique Eparvier

**Affiliations:** 1 CNRS-Institut de Chimie des Substances Naturelles, UPR2301, Université Paris-Saclay, 91198 Gif-sur-Yvette CEDEX, France; morgane.barthelemy@cnrs.fr (M.B.); nicolas.elie@cnrs.fr (N.E.); 2 School of Pharmaceutical Sciences, EPGL, University of Geneva, University of Lausanne, Rue Michel Servet 1, CH-1211 Geneva, Switzerland; leonie.pellissier@unige.ch (L.P.); jean-luc.wolfender@unige.ch (J.-L.W.); 3 Sorbonne Université, CNRS, Laboratoire de Biodiversité et Biotechnologie Microbienne, USR3579, Observatoire Océanologique, 66650 Banyuls-sur-mer, France; didier.stien@cnrs.fr

**Keywords:** *Astrocaryum sciophilum*, endophytes, molecular networking, antibacterial, cytotoxicity, fatty acids

## Abstract

A library of 197 endophytic fungi and bacteria isolated from the Amazonian palm tree *Astrocaryum sciophilum* was extracted and screened for antibacterial activity against methicillin-resistant *Staphylococcus aureus* (MRSA). Four out of five antibacterial ethyl acetate extracts were also cytotoxic for the MRC-5 cells line. Liquid chromatography coupled to tandem mass spectrometry (UPHLC-HRMS/MS) analyses combined with molecular networking data processing were carried out to allow the identification of depsipeptides and cyclopeptides responsible for the cytotoxicity in the dataset. Specific ion clusters from the active *Luteibacter* sp. extract were also highlighted using an MRSA activity filter. A chemical study of *Luteibacter* sp. was conducted leading to the structural characterization of eight fatty acid exhibiting antimicrobial activity against MRSA in the tens of µg/mL range.

## 1. Introduction

Endophytes are microorganisms that live inside the tissues of a host plant without causing apparent symptoms either for all or a part of their life cycle [1]. This colonization is ubiquitous for all plants in various environments. Indeed, endophytes have been isolated not only in environments ranging from tropical to temperate climates but also in extreme habitats [2,3].

Plants are widely explored for new chemical entities with therapeutic purposes. Plant endophytes are increasingly regarded as an important and a relatively underrated source of natural products for drug discovery. Recent surveys suggested that tropical forests harbour several million hyperdiverse endophyte species [4,5] that are expected to lead to the discovery of unique bioactive secondary metabolites [6]. Additionally, because endophytes contribute to the defence of their host, their metabolites are expected to exert a defensive role, for example acting as antimicrobials or pathogen growth-inhibitors [1].

We have pursued the study of the palm tree *Astrocaryum sciophilum* endophytes. *A. sciophilum* grows as an understorey palm throughout the North-East Amazon (Brazil [Pará, Amapá], French Guiana, Surinam, Guyana) [7]. Its development reflects a long-life cycle with a maturation age reaching approximately 170 years. It has periodical leaf production of 16 months on average, allowing the dating of each leaf [8]. Leaves can reach 20 years of age. As a result, endophytes of this model plant are thought to persist for a long time in leaves and thus are able to resist environmental threats. For all of these reasons, *A. sciophilum* endophytes may have developed antimicrobial weaponry to a greater extent than other microbes.

A collection of 197 strains of cultivable endophytes from young (<16 months) and old (2–21 years) healthy leaves collected from 6 specimens of *A. sciophilum* was cultured, extracted and screened towards methicillin-resistant *Staphylococcus aureus* (MRSA). Additionally, the full strain collection was analysed by high-resolution tandem mass spectrometry profiling (HRMS/MS), and the resulting fragmentation data were organized as molecular networks (MNs). Molecular networking approaches allow the organization of untargeted tandem MS datasets according to their spectral similarity, and generate clusters of structurally related metabolites [9]. These approaches have become powerful tools for navigating the chemical space of complex biological systems and can be used to view the chemical constituents of a wide variety of extracts in a single map.

## 2. Results

### 2.1. Biological Activities of Endophyte Extracts

The 197 endophyte isolates (66 bacteria and 131 fungi) were identified, cultivated on solid media and then extracted with ethyl acetate. All of the extracts were tested for antibacterial activity against methicillin-resistant *S. aureus*. Five extracts, four from fungi (BSNB-0575, -0651, -0303 and -0732) and one from bacteria (BSNB -0721) were active (Minimal Inhibitory Concentration (MIC) ≤ 64 µg/mL). The most active extracts arise from endophytic fungi with MIC of 32 (BSNB-0575 and BSNB-0651), 16 (BSNB-0303) and ≤ 8 µg/mL (BSNB-0732) (Table S1). Three of these four fungal extracts come from the *Fusarium* genus (BSNB-0575, -0651 and -0303), and the fourth was identified as *Akanthomyces attenuatus*. Extract obtained from bacteria *Luteibacter* sp. (BSNB-0721) have a MIC value of 64 µg/mL.

The cytotoxicity of all 5 antimicrobial extracts was evaluated using a normal human lung fibroblast MRC-5 cell line. The bacterial extracts of *Luteibacter* sp. (BSNB-0721) was not cytotoxic, with an antibacterial selectivity index above 1 (Table S1).

### 2.2. Molecular Networking

All active and inactive ethyl acetate (EtOAc) extracts were analysed by Ultra-High Performance Liquid Chromatography-High Resolution Mass Spectrometry (UHPLC-HRMS/MS) using the data-dependent acquisition mode. Data were first processed by MZmine 2.33 [10], generated by MetGem [11] and molecular networks (MNs) were visualized using Cytoscape 3.7.0.

Relative quantification of the ions was represented by pie chart diagrams, with their proportions based on the respective areas of the corresponding extracted ion chromatograph (XIC) areas [12]. Targeted analysis of the MN was made possible by colouring the nodes based on the extract’s antibacterial activity, highlighting node clusters among the antibacterial extracts.

**Figure 1:**
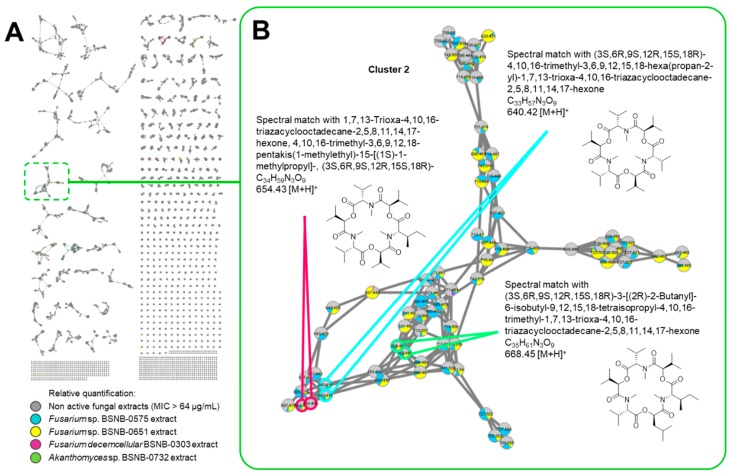
(**A**) Molecular network (MN) of the 131 extracts from fungal endophytes. Relative quantification values of each ion within the extracts are represented as an extracted ion chromatograph (XIC) area-dependent pie chart. (**B**) Dereplication process nodes belongings to cluster 2 composed by ions from the extracts of BSNB-0575 and BSNB-0651. Cluster 2 appears to group the ions corresponding to depsipeptides metabolites. MIC, Minimal Inhibitory Concentration.

A first MN was built with the data from the 131 fungal extracts (Figure S1) composed of 28,719 nodes in 13,060 clusters. Eleven clusters contained nodes from the four active fungal extracts. MS^2^ spectra of the ions at *m/z* 640.42, 654.43, 668.45 (Cluster 2), 600.41, 552.41, 586.39 (Cluster 1) and 730.42 (Cluster 9) matched respectively with three depsipeptides, three pentacyclopeptide and Exumolide A (another depsipeptide) referenced in the available libraries (See Figure 1 and Figure S2). Examination of the MS^2^ spectra of other active clusters showed that these compounds share MS^2^ fragmentation patterns that are similar to that of a peptide because of the typical loss of fragment at *m/z* 113.08 (Leucine/Isoleucine), *m/z* 99.07 (Valine), *m/z* 71.04 (Alanine) and *m/z* 101.05 (Threonine) (See Figure S2). According to the MN, these compounds are also found in extracts with MIC of 128 µg/mL from other *Fusarium* strains. Differences of activity for these extracts can be explained by the relative quantities of metabolites in the extracts (Figure S3).

**Figure 2:**
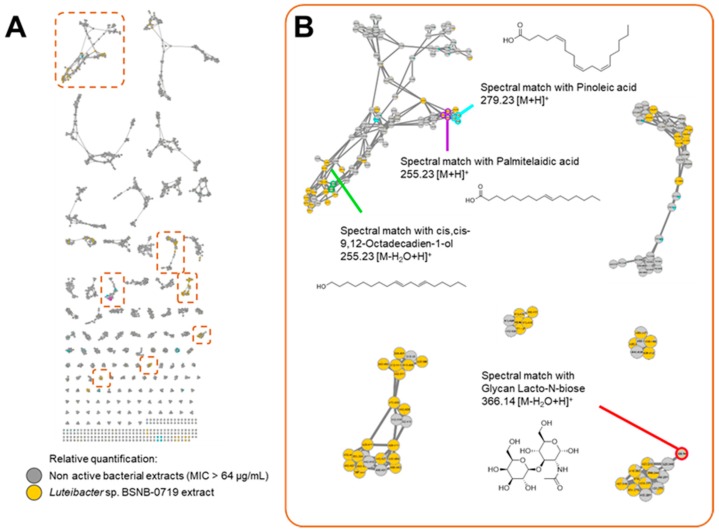
(**A**) Molecular network of the 66 extracts from bacterial endophytes. Relative quantification values of each ion within the extracts are represented as a XIC area-dependent pie chart. (**B**) Clusters of ions from the active extracts of *Luteibacter* sp. BSNB-0721.

The MN generated from the data of the 66 bacterial endophytes extract is composed of 13,763 nodes in 4485 clusters (Figures S4 and S5). This MN showed clusters containing nodes from the one active extract of *Luteibacter* sp. BSNB-0721 (Figure 2A). Comparison of experimental spectral MS^2^ data obtained with databases supported the dereplication of a cluster with fatty acids analogues (Figure 2B). Consequently, the dereplication process could highlight the presence of fatty acid derivatives in *Luteibacter* extract. Other clusters contained nodes specific to the extract of the *Luteibacter* sp. strain and indicated a valuable chemical diversity in this particular strain.

### 2.3. Isolated Compounds from Luteibacter sp. BSNB-0721

The ethyl acetate extract of *Luteibacter* sp. BSNB-0721 was subjected to bioguided fractionation and eight pure compounds were isolated from the three active fractions (See Table S2). All isolated compounds have been determined by spectroscopic data (Figures S6–S29). These compounds were identified as fatty acids with an aliphatic chain of between 15 and 17 carbons that may be terminated with an isopropyl group. Five bear a hydroxyl group on position 2: (*R*)-2-hydroxy-13-methyltetradecanoic acid (1) or on position 3 (*R*)-3-hydroxy-14-methylpentadecanoic acid (2), (*S*)-β-hydroxypalmitic acid (3), (*R*)-3-hydroxy-15-methylhexadecanoic acid (4) and (*R*)-3-hydroxy-13-methyltetradecanoic acid (5). We also isolated and identified 13-methyltetradecanoic acid (6) that does not exhibit a hydroxyl group. The configurations of the compounds were determined by measuring the optical rotation and comparing with the corresponding literature values.

Compounds (7) and (8) presented an unsaturated chain. Based on the coupling constant of the multiplet (δ_H_ 5.35), the *Z* configuration was determined. The localization of the unsaturation was determined by MS^2^ analysis using the method described by Vrkoslav and Cvacka [13]. After esterification, the two molecules were subjected to atmospheric pressure chemical ionization (APCI) leading to the formation of [M+C_3_H_5_N]^+^ adducts. Careful analysis of the MS^2^ data allowed the determination of the double bonds’ position (Figures S30 and S31). These two compounds were finally identified as (9*Z*)-hexadecenoic acid (7) and 15-Methyl-(9*Z*)-hexadecenoic acid (8).

These fatty acids were tested for their antimicrobial activities against MRSA. They showed MIC values ranging from 128 to 32 µg/mL. The most active compound (7) is a C17 fatty acid with a Z double bond at C-9. Fatty acids with hydroxyl at C-3 (2 – 5) or gem-dimethyl at the final position (8) appear to have lower antibacterial activities (Table 1).

## 3. Discussion

Our study, related to the secondary metabolites of the plant host model *Astrocaryum sciophilum*’s associated microorganisms, demonstrated that isolated endophytic strains could produce antibacterial compounds. Indeed, six out of 197 endophytic extracts displayed significant antimicrobial activity.

The four antibacterial extracts derived from fungi (three *Fusarium* strains and one *Akanthomyces* strain) also exhibited cytotoxic activity against MRC-5 cells. Thus, there is a high probability that the antibacterial activity of these extracts depends on the presence of cytotoxic secondary metabolites. Using a molecular networking-based approach, several structurally related analogues of depsipeptide and cyclic peptide - molecules known to be cytotoxic - were highlighted from four active fungal extracts. Depsipeptides have been frequently isolated from fungi belonging to the *Fusarium* genus [14]. More precisely, three cyclic pentapeptides and a cyclic lipopeptide Fusaristatin A were isolated from a strain of *Fusarium decemcellular* [15]. The isolation of a strain from the *Akanthomyces* genus is quite surprising because it is known as a spider parasitic genus. Previous chemical studies of species from this genus have led to the isolation of glycosylated derivatives [16] and pyrone derivatives [17]. Nevertheless, the prediction of numerous peptides in the four active fungal extracts could explain their biological activities. To the best of our knowledge, the present work represents the first time that this class of compounds was detected in the *Akanthomyces* genus.

Of the 66 isolated and extracted bacteria, two shown an antibacterial activity with no cytotoxicity: one *Luteibacter* strain (BSNB-0721) and one *Bacillus* strain (BSNB-0730).

Bacteria from the genus *Luteibacter* were first isolated from the rhizosphere of Barley [18]. To date, three species of this genus have been described: *L. anthropi*, *L. yeojuensis* [19] and *L. rhizovicinus*. No chemical study has ever been performed on this genus, and the analysis of the UHPLC-HRMS/MS data of its EtOAc extract presumed the presence of specific unknown metabolites and fatty acid derivatives. Thus, the extract from the *Luteibacter* sp. (BSNB-0721) was selected for further investigation in order to identify bioactive compounds against MRSA. Eight fatty acid derivatives were isolated in the present study as the major compounds of the EtOAc extract. They appeared to be responsible for the antibacterial activity observed in the crude extract. Nevertheless, ions from the MN cluster unique to the *Luteibacter* extract were not recovered with a fractionation by reverse-phase chromatography because they may be produced in minute amounts and should be further investigated.

The antifungal and bactericidal properties of fatty acids (FA) have been widely studied. Their mechanisms of action are not completely understood; however, it is accepted that FA disrupt cell membranes [20,21]. Many studies have analysed the relationships between FA structure and antibacterial activity, and some general trends have been elucidated. Saturated fatty acids composed of 12 carbons in the chain appear to be the most active, and their activity decreases as the chain becomes either longer or shorter [20,22]. The presence of a *Z* double bond was found to increase the antibacterial activity of a fatty acid, whereas a *trans* configuration leads to an inactive compound [22]. Moreover, a free carboxyl group allows better antibacterial activity than that of esterified fatty acids [23]. These molecules may be an alternative to the common antimicrobial agents for applications in agriculture, food preservation or cosmetics [22,24]. Such literature data may explain, in part, the highest activity observed in this study for compounds 1, 7 and 8. Indeed, it is observed in our study that there is no difference in the activity between unbranched and isobranched FA. The presence of a hydroxyl group at position 3 does not appear to increase the antibacterial activity but may increase the solubility of these compounds. However, in agreement with the literature data, we noticed that the presence of a *Z* double bond enhances antibacterial activity. *Luteibacter*’s ability to produce such a panel of FA must be correlated with its ability for defence against aggressors and must improve its survival. The origin of the FA remains uncertain. In fact, they could be sequestered in the membranes until quick release by some lipases during bacterial attack. Further investigation to identify their biosynthetic origin should be performed later.

## 4. Materials and Methods

### 4.1. General

Optical rotations were measured using an Anton Paar MCP 200 polarimeter (Anton Paar Graz, Austria) in a 350 µL cell with a length of 100 mm. NMR spectra were recorded in CD_3_OD using a Bruker 500 MHz spectrometer or a Bruker 600 MHz spectrometer equipped with a 2 mm invers detection probe (Bruker, Rheinstetten, Germany). Chemical shifts (*δ*) are reported in ppm relative to the TMS (tetramethylsilane) signal. Coupling constants (J) are in Hertz. High-resolution ESITOFMS measurements were performed using a Waters Acquity UPLC system (Waters, Manchester, England) with a column bypass coupled to a Waters Micromass LCT (Low Chromatography Times-of-flight) Premier time-of-flight mass spectrometer equipped with an electrospray interface (ESI). Flash chromatography was performed using a Grace Reveleris system equipped with a 120 g C_18_ column. The flow rate was 80 mL/min, and detection was performed with dual UV at 210 and 270 nm and ELSD (Evaporative Light Scattering Detector). Analytical and preparative HPLC experiments were conducted using a Gilson system equipped with a 322 pumping device, GX-271 fraction collector, 171 diode array detector and prepELSII detector electrospray nebulizer. The columns used for analytical experiments included a Phenomenex Luna C_18_ 5 µm 4.6 × 250 mm and a Phenomenex Luna C_8_ 5 µm 4.6 × 250 mm. The columns used for preparative experiments included a Phenomenex Luna C_18_ 5 µm 21.2 × 250 mm and a Phenomenex Luna C_8_ 5 µm 21.2 × 250 mm (Phenomenex, Le Pecq, France). The flow rates were 1 mL/min and 21 mL/min, respectively, for analytical and preparative experiments and were carried out using a linear gradient of H_2_O mixed with an increasing proportion of acetonitrile (CH_3_CN). Both solvents were modified with 0.1% formic acid. All of the solvents used for analysis were of HPLC grade.

### 4.2. Endophyte Material

*Astrocaryum sciophilum* palm trees were sampled in French Guiana in Piste de Saint-Elie, Sinnamary, in July 2014. The general procedures adopted for the isolation of the microorganisms followed the methodology described by Casella et al. [25]. After collection, the plant material was washed with sterile water and its surface was sterilized by immersion in 70% aqueous ethanol (3 min), followed by immersion in a 5% aqueous sodium hypochlorite (5 min) and finally in 70% aqueous ethanol (1 min). The leaves were cut into small pieces (1–0.5 cm^2^) that were placed in a potato dextrose agar medium (potato dextrose agar (PDA), Fluka Analytical, Steinheim, Germany) in Petri dishes at 28 °C (4–5 parts per Petri dishes). Each individual hyphal tip of emerging fungi was removed and placed in a sterile PDA culture medium in 10 cm Petri dishes. The leaf fragments were cultured for a maximum of 1 month. All of the isolated endophytic strains were deposited in the “ICSN/CNRS Strain Library France”. The strains are maintained in triplicate at −80 °C in a 2 mL cryotube containing 1 mL of a solution of glycerol and water (1:1).

### 4.3. Identification of Endophytic Strains

Fungal and bacterial strains were identified using nucleotides sequencing of rDNA ITS (ITS1-5, 8S-ITS2) and rDNA 16S regions, respectively. The obtained sequences were then submitted to BLAST on NCBI to identify the strain. The sequence data were submitted to GenBank with an accession number for each strain (Table S1).

### 4.4. Cultures and Extraction

Each strain was cultivated at 28 °C in 10 Petri dishes (10 cm diameter) of the PDA culture media. Then, the culture was extracted with ethyl acetate (EtOAc) at room temperature for 24 h. The organic phase was removed via filtration, washed three times with H_2_O, dried with anhydrous solid Na_2_SO_4_ and evaporated using a rotary evaporator under reduced pressure to yield a crude mixture. An extract of the culture media without microorganisms was also conducted.

### 4.5. UPLC-HRMS Analysis

Chromatographic separation was performed using an Acquity UHPLC system interfaced to a Q-Exactive Plus mass spectrometer (Thermo Scientific, Bremen, Germany), using a heated electrospray ionization (HESI-II) source. Thermo Scientific Xcalibur 2.1 software was used for instrument control and data analysis. The LC conditions were as follows: column, Waters BEH (Ethylene Bridget Hybrid) C18 50 × 2.1 mm, 1.7 μm; mobile phase, (A) water with 0.1% formic acid; (B) acetonitrile with 0.1% formic acid; flow rate, 600 μL/min; injection volume, 1 μL; gradient, linear gradient of 5−100% B over 7 min and isocratic at 100% B for 1 min. An Acquity UPLC photodiode array detector was used to acquire the PDA spectra which were collected in the 200–500 nm range. In the positive ion mode, the di-isooctyl phthalate C_24_H_38_O_4_ [M + H]^+^ ion (*m/z* 391.28429) was used as the internal lock mass. The optimized HESI-II parameters were as follows: source voltage, 3.5 kV (pos); sheath gas flow rate (N_2_), 55 units; auxiliary gas flow rate, 15 units; spare gas flow rate, 3.0; capillary temperature, 275.00 °C (pos), S-Lens RF Level, 45. The mass analyser was calibrated using a mixture of caffeine, methionine−arginine− phenylalanine−alanine−acetate (MRFA), sodium dodecyl sulphate, sodium taurocholate, and Ultramark 1621 in an acetonitrile/ methanol/water solution containing 1% formic acid by direct injection. The data-dependent MS/MS events were performed on the four most intense ions detected in full scan MS (Top3 experiment). The MS/MS isolation window width was 1 Da, and the normalized collision energy (NCE) was set to 35 units. In the data-dependent MS/MS experiments, full scans were acquired at a resolution of 35,000 FWHM (at *m/z* 200) and MS/MS scans were acquired at 17,500 FWHM both with a maximum injection time of 50 ms. After being acquired in a MS/MS scan, the parent ions were placed on the dynamic exclusion list for 2.0 s.

### 4.6. MZmine 2.33 Data-Preprocessing Parameters

Raw files were converted into MzXML (mass spectrometry data format) files using the MSConvert software. Then, MzXML files were processed using Mzmine 2.37 [10]. Mass detection was carried out with a centroid mass detector with the noise level set to 5.0E5 for the MS level set to all. The ADAP (Automated Data Analysis Pipeline) chromatogram builder [26] was achieved using a minimum group size of scans of 5, a minimum group intensity threshold of 5.0E5, a minimum highest intensity of 5.0E5 and a *m/z* tolerance of 0.002 or 5 ppm. The wavelets (ADAP) algorithm was used for the chromatogram deconvolution with the following settings: S/N threshold of 10, an intensity window SN, a minimum feature height of 1000, a coefficient area threshold of 100, a peak duration range between 0.01 and 0.5 and an RT wavelet range between 0.001 and 0.05. The *m/z* and RT range for MS^2^ scan pairing were set to 0.001 Da and 0.3 min, respectively. Chromatograms were deisotoped using the isotopic peaks grouper algorithm with an *m/z* tolerance of 0.003 (5 ppm), RT tolerance of 0.1 (absolute), maximum charge of 2 and the representative isotope used was the most intense. Peak alignment was performed using the join aligner method: *m/z* tolerance of 0.001 or 5.0 ppm, weight for *m/z* of 0.001, RT tolerance of 0.3 min, weight for RT of 0.1. Adduct search (Na^+^, K^+^, NH_4_^+^, ACN^+^) was conducted on the peak list with an RT tolerance set to 1.0 min and the maximum relative peak height at 50%. The found adducts were then removed from the peak list. The peak list was gap-filled with the peak finder module: intensity tolerance of 90%, *m/z* tolerance of 0.001 or 5.0 ppm and RT tolerance of 0.1 min. For further analysis, the peak list was reduced to the ions with the *m/z* values between 200 and 900, in order to decrease the number of data.

### 4.7. Molecular Network Analysis

After preprocessing the UHPLC-HRMS/MS data with MZmine 2.33, the output mgf file was processed with the MetGem software [11] to give a network containing nodes distributed in clusters. To decrease the size of the peak list, ions with the *m/z* values of 200–900 and/or selfloop nodes can be removed. Networks were generated using the following parameters: *m/z* tolerance set to 0.02, Minimum Matched Peaks set to 6, topK set to 10, Minimal Cosine score Value of 0.7 and Max. Connected Component Size of 100. Then, the associated CSV file was loaded. For the mapping process, the relative quantification of each ion was represented by pie chart-diagrams for which the proportions were based on the respective areas of the corresponding extracted ion chromatograph areas (XIC). Then, the analogues of the spectra in the network were searched in the available spectral libraries. The library spectra were filtered in the same manner as the input data. All of the matches between network spectra and library spectra were required to have a score above 0.7 and at least 6 matched peaks. The *m/z* tolerance for the analogues’ search was set to 100. For a more advanced retreatment of the network, the MN was also exported to the Cytoscape 3.7.0 software (https://cytoscape.org/).

### 4.8. Large-Scale Cultivation of Luteibacter sp. and Isolation

Strains were cultivated 15 days at 28 °C in 14 cm Petri dishes of PDA (potato dextrose agar) media. Culture media was extracted three times consecutively with ethyl acetate (EtOAc) at room temperature (the organic phase after 24 h and replacing the remaining agar in EtOAc). The combined organic solution was washed as described above.

Large-scale cultivation of *Luteibacter* sp. was conducted on 210 14 cm Petri dishes to yield 1.96 g of a brown-yellow crude extract. This crude extract (1.8 g) was fractionated by reverse flash chromatography on a C_18_ column with a 5-min-step gradient of water mixed with an increasing proportion of acetonitrile (*v/v*, 95:5, 75:25, 50:50, 20:80 and 0:100). Six fractions were generated based on the UV and ELSD detection: F1 (22.2 mg, 1.2%), F2 (165.5 mg, 9.2%), F3 (93.6 mg, 5.2%), F4 (41.0 mg, 2.3%), F5 (46.3 mg, 2.6%) and F6 (563.8 mg, 31.3%). A step gradient of acetonitrile—methylene chloride (*v/v*, 50:50–0:100) was conducted to generate 2 additional fractions: F7 (334.4 mg, 18.6%), F8 (91.8 mg, 5.1%).

F4 (35.0 mg) was purified by preparative HPLC (Luna C_18_, mobile phase H_2_O + 0.1% FA/CH_3_CN + 0.1% FA, isocratic elution 60:40 during 6 min then linear gradient from 60:40 to 0:100 over 13 min, flow rate 21 mL/min) to obtain *(R)*-3-hydroxy-13-methyltetradecanoic acid (5) (0.2 mg, t_R_ = 26.5 min). F5 (41.4 mg) was purified by preparative HPLC (Luna C_8_, mobile phase H_2_O + 0.1% FA / CH_3_CN + 0.1% FA, isocratic elution 40:60 during 6 min then linear gradient from 40:60 to 0:100 over 13 min, flow rate 21 m/min) to obtain *(R)*-2-hydroxy-13-methyltetradecanoic acid (1) (3.7 mg, t_R_ =14.0 min), *(R)*-3-hydroxy-14-methylpentadecanoic acid (2) (1.5 mg, t_R_ = 15.3 min), *(S)*-β-hydroxypalmitic acid (3) (1.9 mg, t_R_= 15.6 min) and 9*Z*-hexadecenoic acid (7) (0.4 mg, t_R_= 21.5 min). F6 (258.0 mg) was purified by preparative HPLC (Luna C_8_, mobile phase H_2_O + 0.1% FA / CH_3_CN + 0.1% FA, isocratic elution 35:65 during 30 min, flow rate 21 mL/min) to obtain *(R)*-3-hydroxy-15-methylhexadecanoic acid (4) (7.2 mg, t_R_ = 15.0 min), 9*Z*-hexadecenoic acid (7) (30.7 mg, t_R_ = 21.0 min), 13-methyltetradecanoic acid (6) (62.2 mg, t_R_ = 24.0 min) and 15-methyl-9*Z*-hexadecenoic acid (8) (63.8 mg, t_R_ = 27.0 min).

**(*R*)-2-hydroxy-13-methyltetradecanoic acid (1)**: White powder. [α]_D_^20^ = −5.6 (*c* = 0.5, chloroform). ^1^H-NMR (500 MHz, CD_3_OD): 4.07 (1H, *dd*, *J* = 7.4, 4.4, H-2), 1.75 (1H, *m*, H-3), 1.63 (1H, *m*, H-3), 1.52 (1H, *non*, *J* = 6.7, H-3), 1.44 (2H, *m*, H-4), 1.30 (14H, *s,* H-5 to H-11), 1.17 (2H, *m*, H-11), 0.88 (6H, *d*, *J* = 6.8, H-14, H-15). ^13^C-NMR (500 MHz, CD_3_OD): 178.6 (C-1), 71.9 (C-2), 40.4 (C-12), 35.7 (C-3), 31.2–30.8 (5C, C-6 to C-10), 30.7 (C-5), 29.3 (C-13), 28.7 (C-11), 26.3 (C-4), 23.2 (2C, C-14, C-15). HR-ESI-MS: 257.2109 ([M − H]^−^, C_15_H_29_O_3_^−^; calc. 257.2122), 515.4294 ([2M − H]^−^, C_30_H_59_O_6_^−^: calc. 515.4317).

**(*R*)-3-hydroxy-14-methylpentadecanoic acid (2)**: White powder. [α]_D_^20^ = −5.6 (c = 0.5, chloroform). ^1^H-NMR (600 MHz, CD_3_OD): 3.96 (1H, *m*, H-3), 2.42 (1H, *dd*, *J* = 15.1, 4.7, H-2), 2.34 (1H, *dd*, *J* = 15.1, 8.2, H-2), 1.52 (1H, *non*, *J* = 6.7, H-14), 1.47 (4H, *m*, H-4, H-5), 1.30 (14H, *s*, H-5 to H-12), 1.18 (2H, *m*, H-13), 0.88 (6H, *d*, *J* = 6.8, H-15, H-16). ^13^C-NMR (600 MHz, CD_3_OD): 176.7 (C-1), 69.6 (C-3), 43.7 (C-2), 40.4 (C-13), 38.2 (C-4), 31.2-30.9 (6C, C-6 to C-11), 29.3 (C-14), 28.7 (C-12), 26.8 (C-5), 23.2 (2C, C-15, C-16). HR-ESI-MS: 271.2274 ([M − H]^−^, C_16_H_31_O_3_^−^; calc. 271.2279), 543.4611 ([2M − H]^−^, C_32_H_63_O_6_^−^; calc. 543.4630).

**(*S*)-*β*-hydroxypalmitic acid (3)**: White powder. [α]_D_^20^ = +18 (c = 0.1, chloroform). ^1^H-NMR (600 MHz, CD_3_OD): 3.97 (1H, *m*, H-3), 2.43 (1H, *dd*, *J* = 15.1, 4.7, H-2), 2.36 (1H, *dd*, *J* = 15.1, 8.2, H-2), 1.47 (4H, *m*, H-4, H-5), 1.30 (20H, *s*, H-6 to H-15), 0.90 (3H, *t*, *J* = 7.2, H-16). ^13^C-NMR (600 MHz, CD_3_OD): 176.1 (C-1), 69.5 (C-3), 43.5 (C-2), 38.3 (C-4), 33.2 (C-14), 30.9-30.6 (8C, C-6 to C-13), 26.8 (C-5), 23.9 (C-15), 14.6 (C-16). HR-ESI-MS: 271.2274 ([M − H]^-^, C_16_H_31_O_3_^-^; calc. 271.2279), 543.4611 ([2M − H]^−^, C_32_H_63_O_6_^−^; calc. 543.4630).

**(*R*)-3-hydroxy-15-methylhexadecanoic acid (4)**: White powder. [α]_D_^20^ = −20 (c = 0.1, chloroform). ^1^H-NMR (500 MHz, CD_3_OD): 3.96 (1H, *m*, H-3), 2.43 (1H, *dd*, *J* = 15.1, 4.7, H-2), 2.35 (1H, *dd*, *J* = 15.2, 8.2, H-2), 1.53 (1H, *non*, *J* = 6.7, H-15), 1.47 (2H, *m*, H-4), 1.30 (18H, *s*, H-5 to H-12), 1.18 (2H, *m*, H-14), 0.88 (6H, *d*, *J* = 6.6, H-16, H-17). ^13^C-NMR (500 MHz, CD_3_OD): 176.5 (C-1), 69.7 (C-3), 43.7 (C-2), 40.4 (C-14), 38.3 (C-4), 31.2-30.9 (7C, C-6 to C-12), 29.3 (C-15), 28.7 (C-12), 26.8 (C-5), 23.2 (2C, C-15, C-16). HR-ESI-MS: 285.2431 ([M − H]^−^, C_17_H_33_O_3_^−^; calc. 285.2435), 571.4926 ([2M − H]−^,^ C_34_H_67_O_6_^−^; calc. 571.4943).

**(*R*)-3-hydroxy-15-methylhexadecanoic acid (4)**: White powder. [α]_D_^20^ = −20 (c = 0.1, chloroform). ^1^H-NMR (500 MHz, CD_3_OD): 3.96 (1H, *m*, H-3), 2.43 (1H, *dd*, *J* = 15.1, 4.7, H-2), 2.35 (1H, *dd*, *J* = 15.2, 8.2, H-2), 1.53 (1H, *non*, *J* = 6.7, H-15), 1.47 (2H, *m*, H-4), 1.30 (18H, *s*, H-5 to H-12), 1.18 (2H, *m*, H-14), 0.88 (6H, *d*, *J* = 6.6, H-16, H-17). ^13^C-NMR (500 MHz, CD_3_OD): 176.5 (C-1), 69.7 (C-3), 43.7 (C-2), 40.4 (C-14), 38.3 (C-4), 31.2-30.9 (7C, C-6 to C-12), 29.3 (C-15), 28.7 (C-12), 26.8 (C-5), 23.2 (2C, C-15, C-16). HR-ESI-MS: 285.2431 ([M − H]^−^, C_17_H_33_O_3_^−^; calc. 285.2435), 571.4926 ([2M − H]−^,^ C_34_H_67_O_6_^−^; calc. 571.4943).

**(*R*)-3-hydroxy-13-methyltetradecanoic acid (5)**: White powder. [α]_D_^20^ = −15 (c = 0.1, chloroform). ^1^H-NMR (600 MHz, CD_3_OD): 3.89 (1H, *s*, H-3), 2.33 (1H, *m*, H-2), 2.24 (1H, *m*, H-2), 1.52 (1H, *m*, H-13), 1.45 (4H, *s*, H-4, H-5), 1.30 (12H, *s*), 1.18 (2H, *s*, H-12), 0.88 (6H, *d*, *J* = 6.6, H-14, H-15). ^13^C-NMR (600 MHz, CD_3_OD): 176.5 (C-1), 70.4 (C-3), 45.3 (C-2), 40.4 (C-12), 38.2 (C-4), 31.2-30.9 (5C, C-6 to C-10), 29.3 (C-13), 28.7 (C-11), 26.8 (C-5), 23.1 (2C, C-14, C-15). HR-ESI-MS: 257.2101 ([M − H]^−^, C_15_H_29_O_3_^−^; calc. 257.2122), 515.4285 ([2M − H]^−^, C_30_H_59_O_6_^−^; calc. 515.4317).

**13-methyltetradecanoic acid (6)**: White powder. ^1^H-NMR (500 MHz, CD_3_OD): 2.27 (1H, t, *J* = 7.4, H-2), 1.60 (1H, m, H-3), 1.52 (1H, *non*, *J* = 6.6, H-13), 1.30 (16H, s, H-4 to H-11), 1.18 (1H, m, H-12), 0.88 (6H, d, *J* = 6.7, H-14, H-15). ^13^C-NMR (500 MHz, CD_3_OD): 177.9 (C-1), 40.4 (C-11), 35.2 (C-2), 31.2-30.4 and 28.7 (8C, C-4 to C-10), 29.3 (C-13), 26.3 (C-3), 23.2 (2C, C-14, C-15). HR-ESI-MS: 241.2168 ([M − H]^−^, C_15_H_29_O_2_^−^; calc. 241.2173), 483.4432 ([2M − H]^−^, C_30_H_59_O_4_^−^; calc. 483.4419).

**9*Z*-hexadecenoic acid (7)**: yellow oily liquid. ^1^H-NMR (500 MHz, CD_3_OD): 5.35 (2H, dt, *J* = 11.3, 6.2, H-9, H-10), 2.27 (2H, t, *J* = 7.5, C-2), 2.03 (4H, m, H-8, H-11), 1.60 (2H, m, H-3), 1.33 (16H, s), 0.90 (3H, t, *J* = 6.9, H-16). ^13^C-NMR (500 MHz, CD_3_OD): 178.0 (C-1), 131.1 (C-10), 130.9 (C-11), 35.3 (C-2), 33.1 (C-14), 31.0 (*s*, C-13), 31.0-30.2 (5C, C-4 to C-8), 28.3 (2C, C-9, C-12), 26.3 (C-3), 23.9 (C-15), 14.6 (C-16). HR-ESI-MS: 255.2326 ([M + H]^+^, C_16_H_31_O_2_^+^; calc. 255.2319), 253.2164 ([M − H]^−^, C_16_H_29_O_2_^−^; calc. 253.2162), 507.4426 ([2M − H]^−^, C_32_H_59_O_4_^−^; calc. 507.4408).

**15-methyl-9*Z*-hexadecenoic acid (8)**: yellow oily liquid. ^1^H-NMR (500 MHz, CD_3_OD): 5.35 (2H, dt, *J* = 11.3, 6.2, H-10, H-11), 2.27 (2H, t, *J* = 7.4, H-2), 2.04 (4H, m, H-9, H-12), 1.60 (2H, m, H-3), 1.53 (1H, *non*, *J* = 6.7, H-15), 1.33 (12H, s), 1.19 (2H, m, H-14), 0.88 (6H, d, *J* = 6.6, H-16, H-17). ^13^C-NMR (500 MHz, CD_3_OD): 177.9 (C-1), 131.0 (2C, C-10, C-11), 40.3 (C-14), 35.2 (C-2), 31.3-30.3 (5C, C-4 to C-8), 29.3 (C-15), 28.3 (2C, C-9, C-11), 28.3 (C-13), 26.3 (C-3), 23.2 (2C, C-16, C-17). HR-ESI-MS: 269.2484 ([M + H]^+^, C_17_H_33_O_2_^+^; calc. 269.2475), 267.2334 ([M − H]^−^, C_17_H_31_O_2_^−^; calc. 267.2330), 535.4756 ([2M − H]^−^, C_16_H_31_O_2_^−^; calc. 535.4721).

### 4.9. Preparation of Fatty Acid Methyl Esters (FAMEs)

Preparation of FAMEs was carried out based on methanolysis/methylation using conc. Hydrochloric acid (HCl) as described by Ichihara and Fukubayashi [27]. A solution of 8.0% (*w/v*) HCl was obtained by diluting conc. HCl (37%, *w/w*; 9.1 mL) in methanol (40.9 mL). Each fatty acid was dissolved in toluene to reach a concentration of 0.005 g/mL. Then, methanol (7.5-fold) and 8.0% HCl solution (1.5-fold) were added sequentially to this solution. The solution was stirred at 45°C overnight. After cooling at room temperature, hexane (5-fold) and water (5-fold) were added for the extraction of FAMEs. HCl (37%, *w/w*) was purchased from Carlo Erba^©^.

FAMEs were then diluted in CH_2_Cl_2_ to a concentration of 2 mg/mL and then were diluted in CH_3_CN to the concentration 200 µg/mL. These solutions were analysed by MS/MS on a Q-ToF 6540 mass spectrometer (Agilent, Les Ulis, France) by direct introduction at a flow rate of 10 µL/min and using an APCI (Atmospheric Pressure Chemical Ionization) ion source in the positive mode. The corona current was set to 2 µA, the nebulizer pressure was 60 psig and 8 L/min nitrogen flow heated at 300 °C was used for desolvation. Capillary, fragmentor and skimmer voltages were set to 3000 V, 100 V, and 45 V, respectively. CH_2_Cl_2_ and CH_3_CN were purchased from J.T. Baker^©^. The MS/MS collision energy was 15 (arbitrary units).

### 4.10. Determination of Minimal Inhibitory Concentration

The ATCC strains were purchased from the Pasteur Institute. The strain used in this study was methicillin-resistant *S. aureus* ATCC33591. Extracts, fractions and pure compounds were tested according to the reference protocol of the European Committee on Antimicrobial Susceptibility Testing [28]. The standard microdilution test as described by the Clinical and Laboratory Standards Institute guidelines (M7-A8) was used to determine minimal inhibition concentrations (MIC) against bacteria [29]. Crude extracts and pure compounds were tested at concentrations ranging from 256 to 0.5 μg/mL. The microplates were incubated at 35 °C, and MIC values were calculated after 24 h. The MIC values reported in Table S2 refer to the lowest concentration preventing visible growth in the wells. All assays were conducted in triplicate.

### 4.11. Cytotoxicity Evaluation

The crude extract for each strain was tested to determine its cytotoxicity using the MRC-5 (ATCC CCL-171) normal human lung fibroblast cells (LGC standards, Molsheim, France). MRC-5 cells were seeded into 96-well microplates at 2000 cells per well. The assay was conducted according to the procedure described by Tempête et al. [30]. After 24 h in wells, solubilized extracts in DMSO are deposited in triplicate as well as the solvent and docetaxel (Taxotère) controls. Cell viability is evaluated in comparison with untreated control cultures after 3 days. Extracts are tested at the concentration of 10 μg/mL.

## Figures and Tables

**Table 1:** Minimal Inhibitory Concentration (MIC) of compounds 1–8 against methicillin-resistant *S. aureus*.

	Fatty Acid	MIC on MRSA ^1^ (µg/mL)
**1**	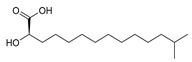	64
**2**	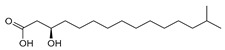	128
**3**	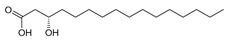	128
**4**	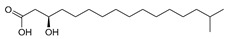	128
**5**	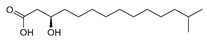	ND
**6**	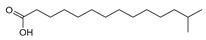	128
**7**	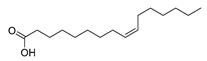	32
**8**	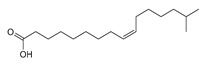	64

^1^ Positive Control: Vancomycin (MIC = 1 µg/mL). ND = Not Determined. MRSA = methicillin-resistant *Staphylococcus aureus*.

## Supplementary Materials

Supplementary materials can be found at https://www.mdpi.com/1422-0067/20/8/2006/s1.

## Abbreviations

MRSA	Methicillin-resistant *Staphylococcus aureus*
UHPLC-HRMS/MS	Ultra-High Performance Liquid Chromatography-High Resolution Mass Spectrometry
MIC	Minimal Inhibitory Concentration
EtOAc	Ethyl acetate
MN	Molecular Network
XIC	Extracted Ion Chromatogram
PDA	Potato Dextrose Agar
FAME	Fatty Acid Methyl Ester
FA	Formic Acid
HCl	Hydrochloric acid
