# Supplementary material for: Structural Identification of Antibacterial Lipids from Amazonian Palm Tree Endophytes through the Molecular Network Approach
Source: Int J Mol Sci. 2019 Apr 24;20(8):2006. doi: 10.3390/ijms20082006 (PMC6514718; doi:10.3390/ijms20082006)
Supplement: Supplementary file 1 [file ijms-20-02006-s001.pdf]

SUPPORTING INFORMATION FOR :

Structural identification of antibacterial lipids from Amazonian palm tree endophytes through the Molecular Network approach

Morgane Barthélemy [1], Nicolas Elie [1], Léonie Pellissier [2], Jean-Luc Wolfender [2], Didier Stien [3], David Touboul [1] and Véronique Eparvier [1,*]

[1] CNRS-Institut de Chimie des Substances Naturelles, UPR2301, Université Paris-Saclay, 91198 Gif-sur-Yvette cedex, France; morgane.barthelemy@cnrs.fr, nicolas.elie@cnrs.fr
[2] School of Pharmaceutical Sciences, EPGL, University of Geneva, University of Lausanne, Rue Michel Servet 1, CH-1211, Geneva, Switzerland; leonie.pellissier@unige.ch, jean-luc.wolfender@unige.ch
[3] Sorbonne Universités, UPMC Univ. Paris 06, CNRS, Laboratoire de Biodiversité et Biotechnologies Microbiennes (LBBM), Observatoire Océanologique, 66650 Banyuls-sur-Mer, France; didier.stien@©cnrs.fr
* Correspondence: veronique.eparvier@cnrs.fr; Tel.: +33-(0)16-982-3679, david.touboul@cnrs.fr; Tel: +33-(0)16-982-3032

Table of content

Molecular network of the 131 extracts from fungal endophytes	S1
Dereplication process and MS^2 fragmentation spectra of nodes belongings to the clusters composed by ions from the four active fungal extracts.	S2
Molecular network of the 66 extracts from bacterial endophytes	S3
Dereplication process of nodes belongings to the clusters composed by ions from the *Luteibacter* sp. extract.	S4
Dereplication process of nodes belongings to the clusters composed by ions from the *Bacillus* sp. extract	S5
NMR spectra and HRMS of *(R)*-2-hydroxy-13-methyltetradecanoic acid **(1)**	S6-S8
NMR spectra and HRMS of *(R)*-3-hydroxy-14-methyl-pentadecanoic acid **(2)**	S9-S11
NMR spectra and HRMS of *(S)*-β-hydroxypalmitic acid **(3)**	S12-S14
NMR spectra and HRMS of *(R)*-3-hydroxy-15-methylhexadecanoic acid **(4)**	S15-17
NMR spectra and HRMS of *(R)*-3-hydroxy-13-methyltetradecanoic acid **(5)**	S18-20
NMR spectra and HRMS of 13-methyltetradecanoic acid **(6)**	S21-23
NMR spectra and HRMS of 9Z-hexadecenoic acid **(7)**	S24-26
NMR spectra and HRMS of 15-methyl-9Z- hexadecenoic acid compound **(8)**	S27-29
Fragmentation of 9Z-hexadecenoic acid **(7)**	S30
Fragmentation of 15-methyl-9Z-hexadecenoic acid **(8)**	S31
Identification of endophytic microorganisms associated with *Astrocaryum sciophilum* and biological activities of their respective EtOAc extracts: antibacterial on methicillin-resistant *S. aureus* (MIC in µg/ml) and cytotoxicity on MRC-5 cells (viability percentage)	**Table S1**
Antibacterial activity of AcOEt extract of *Luteibacter* sp. and its fractions on MRSA (Minimum Inhibitory Concentration in µg/ml)	**Table S2**

Figure S1. Molecular network of the 131 extracts from fungal endophytes. Relative quantification of each ion within the extracts are represented as a XIC area-dependent pie-chart drawing.

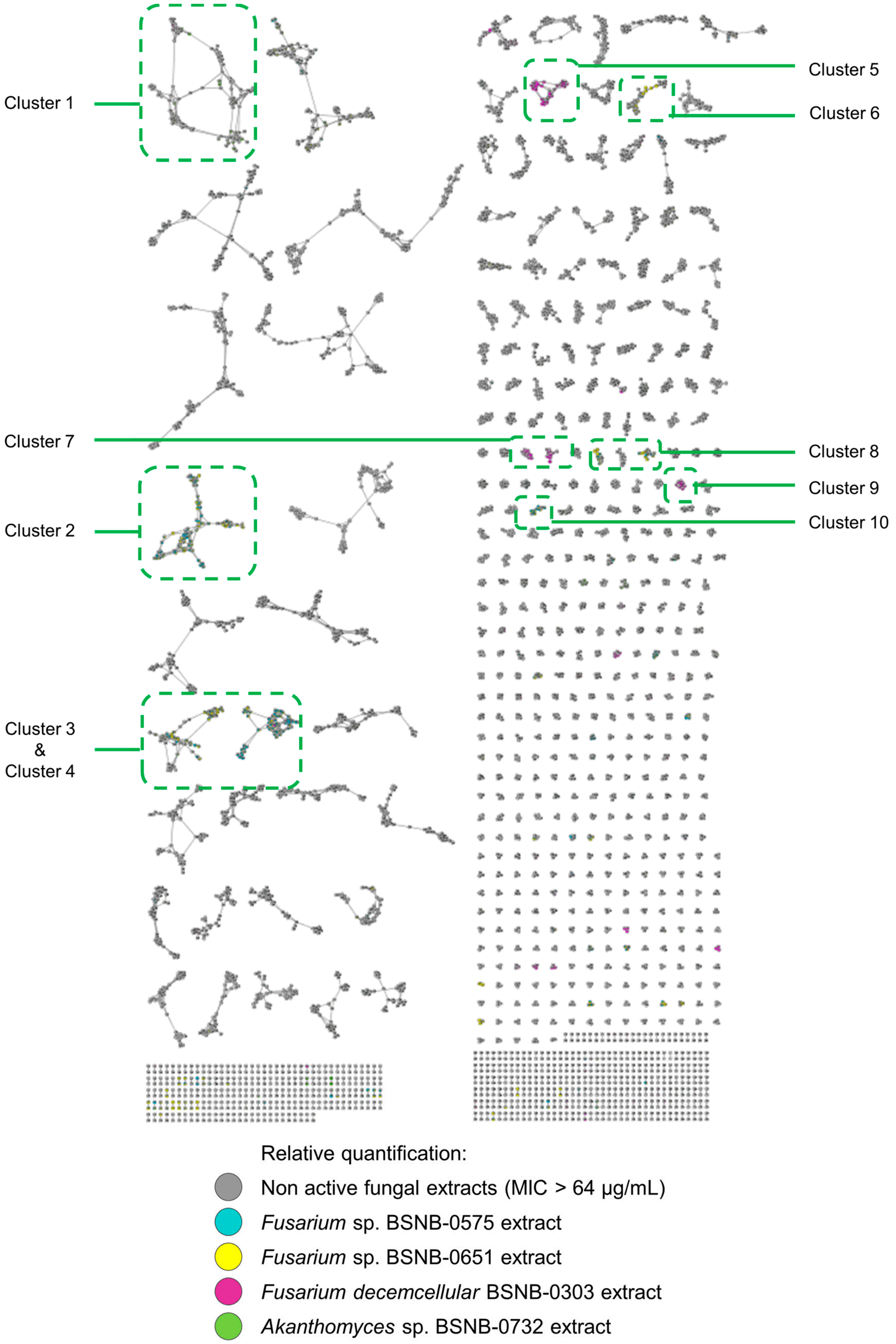

Figure S2. Dereplication process and MS/MS fragmentation spectra of nodes belongings to the clusters composed by ions from the four active fungal extracts

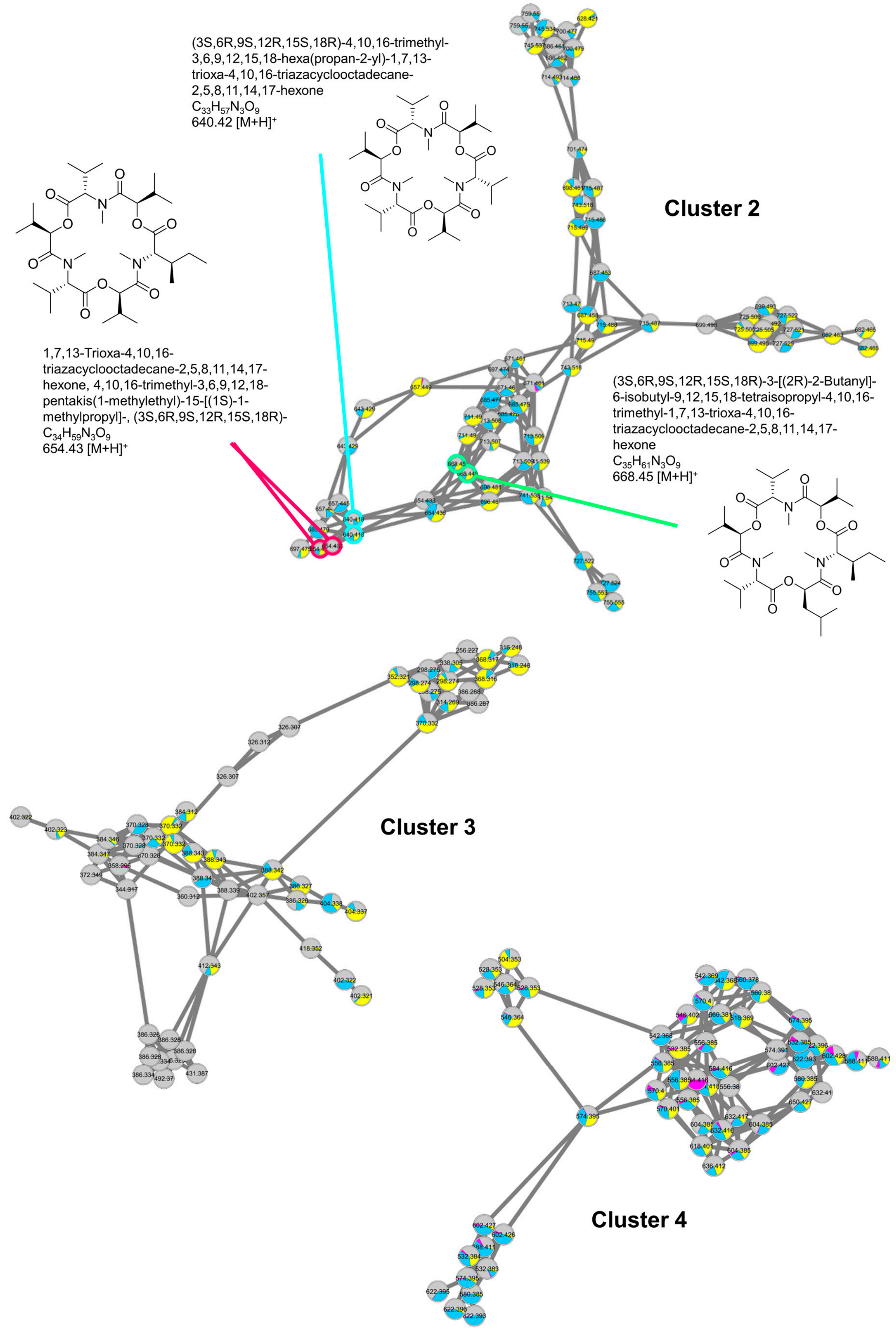

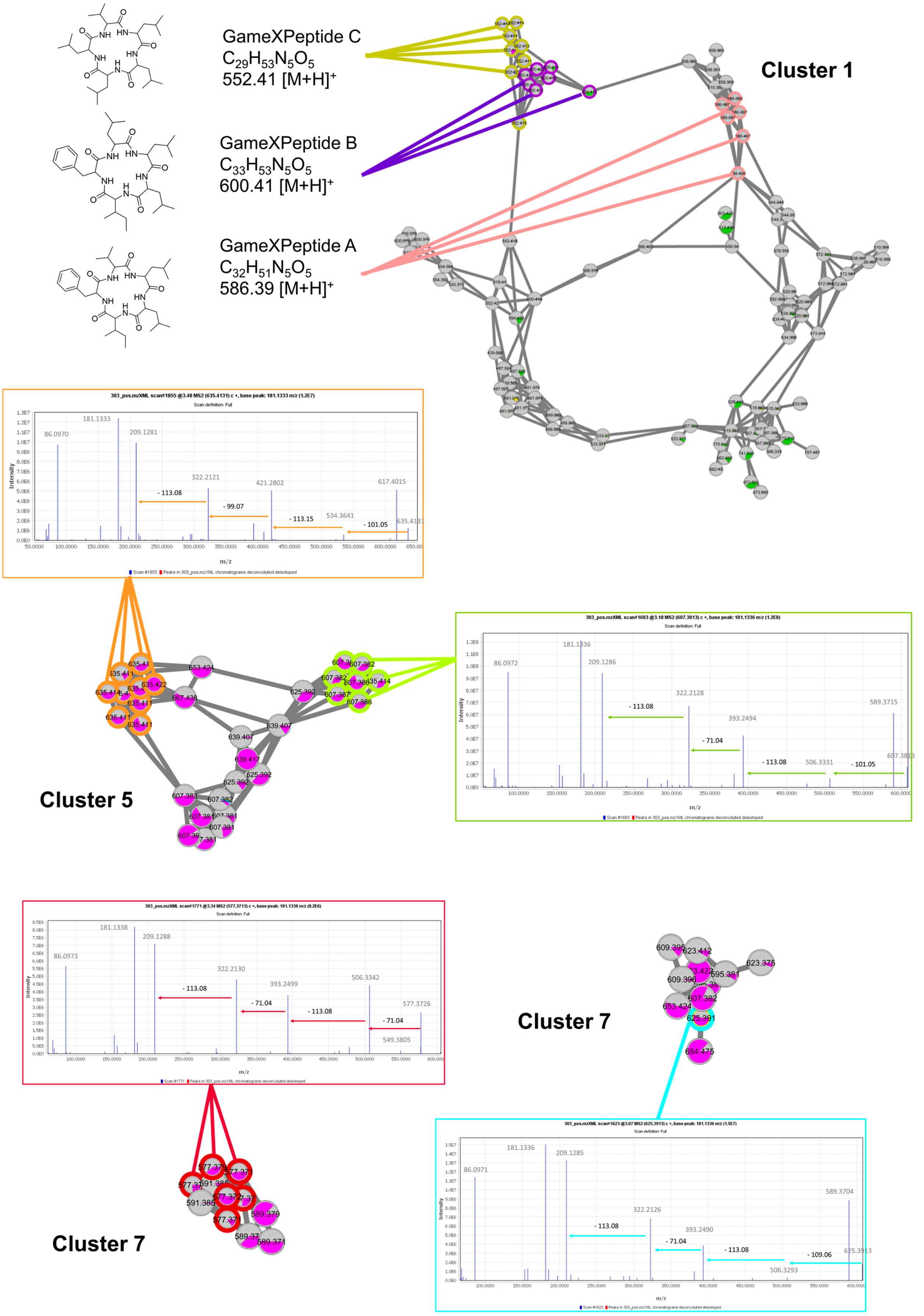
GameXPeptide C
$C_{29}H_{53}N_5O_5$
552.41 $[M+H]^+$
GameXPeptide B
$C_{33}H_{53}N_5O_5$
600.41 $[M+H]^+$
GameXPeptide A
$C_{32}H_{51}N_5O_5$
586.39 $[M+H]^+$
Cluster 1
Cluster 5
Cluster 7
Cluster 7
303_pos.mzXML scan#1855 @3.48 MS2 (635.4131) c +, base peak: 181.1333 m/z (1.2E7)
181.1333
86.0970
209.1281
322.2121
421.2802
617.4015
534.3641
635.4131
- 113.08
- 99.07
- 113.15
- 101.05
303_pos.mzXML scan#1683 @3.18 MS2 (607.3813) c +, base peak: 181.1336 m/z (1.2E8)
181.1336
86.0972
209.1286
322.2128
393.2494
589.3715
506.3331
607.3813
- 113.08
- 71.04
- 113.08
- 101.05
303_pos.mzXML scan#1771 @3.34 MS2 (577.3713) c +, base peak: 181.1338 m/z (8.2E6)
181.1338
209.1288
86.0973
322.2130
393.2499
506.3342
577.3726
549.3805
- 113.08
- 71.04
- 113.08
- 71.04
303_pos.mzXML scan#1623 @3.07 MS2 (625.3913) c +, base peak: 181.1336 m/z (1.5E7)
181.1336
209.1285
86.0971
589.3704
322.2126
393.2490
625.3913
506.3293
- 113.08
- 71.04
- 113.08
- 109.06
m/z
Intensity

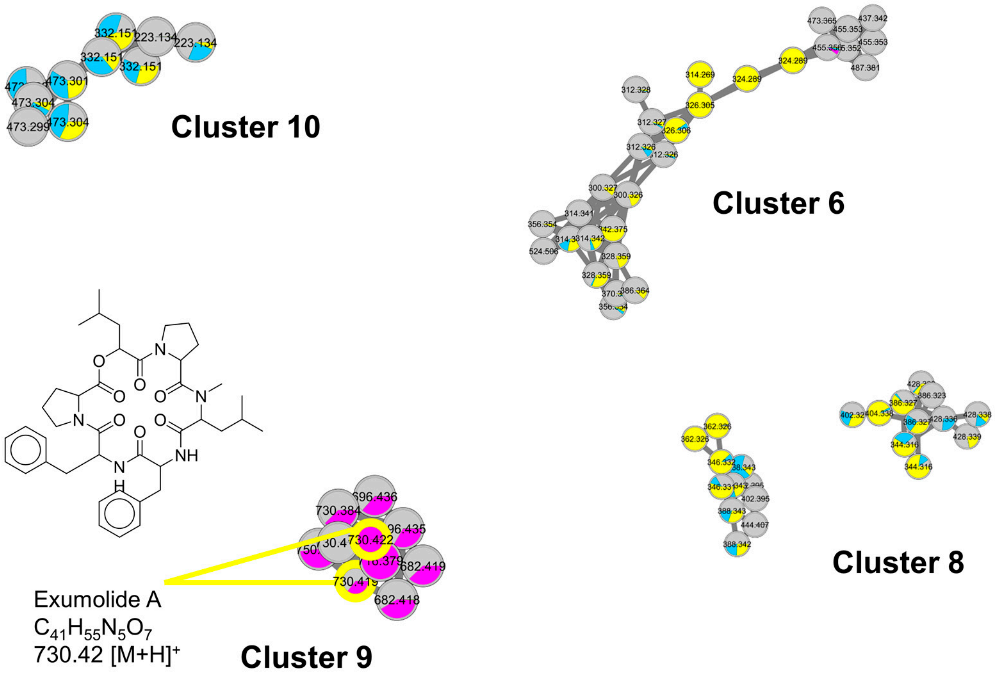
Cluster 10
Cluster 6
Cluster 8
Cluster 9
Exumolide A
$C_{41}H_{55}N_5O_7$
730.42 $[M+H]^+$

Figure S3. Molecular network of the 66 extracts from bacterial endophytes. Relative quantification of each ion within the extracts are represented as a XIC area-dependent pie chart drawing.

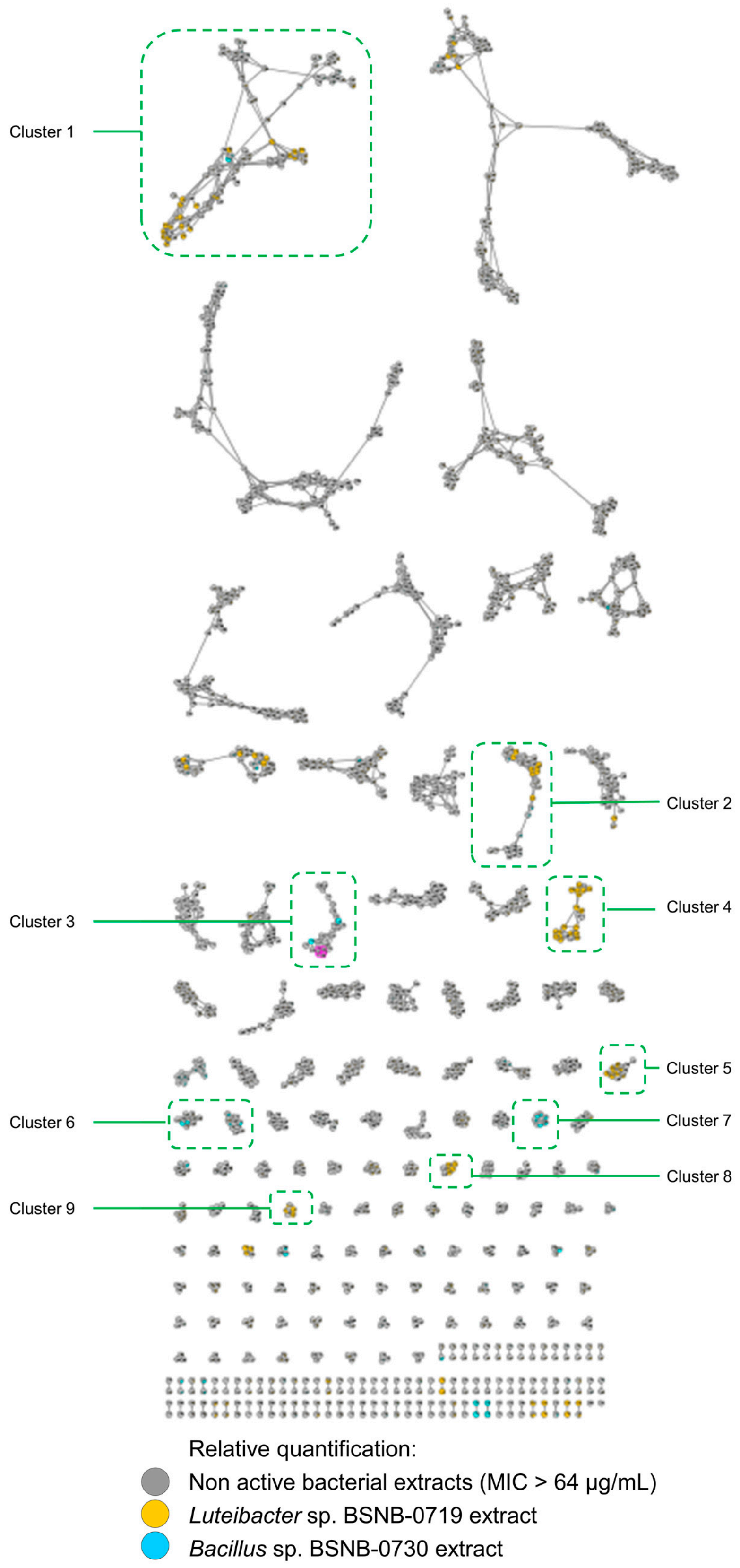

Figure S4. Dereplication process of nodes belongings to the clusters composed by ions from the *Luteibacter* sp. extract.

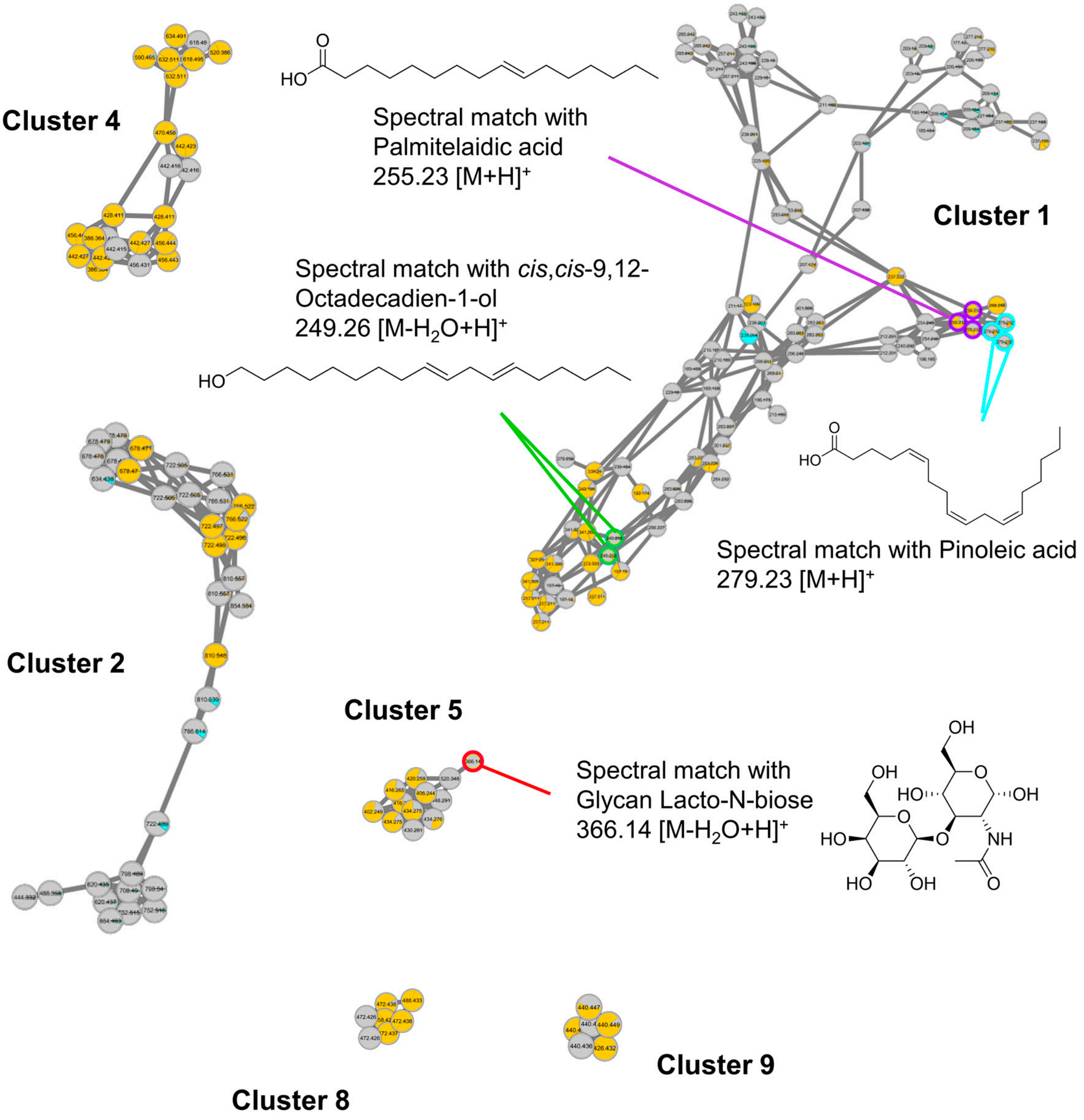

Figure S5. Dereplication process of nodes belongings to the clusters composed by ions from the *Bacillus* sp. extract (left). Relative quantification of those clusters depending on the bacterial genus (right).

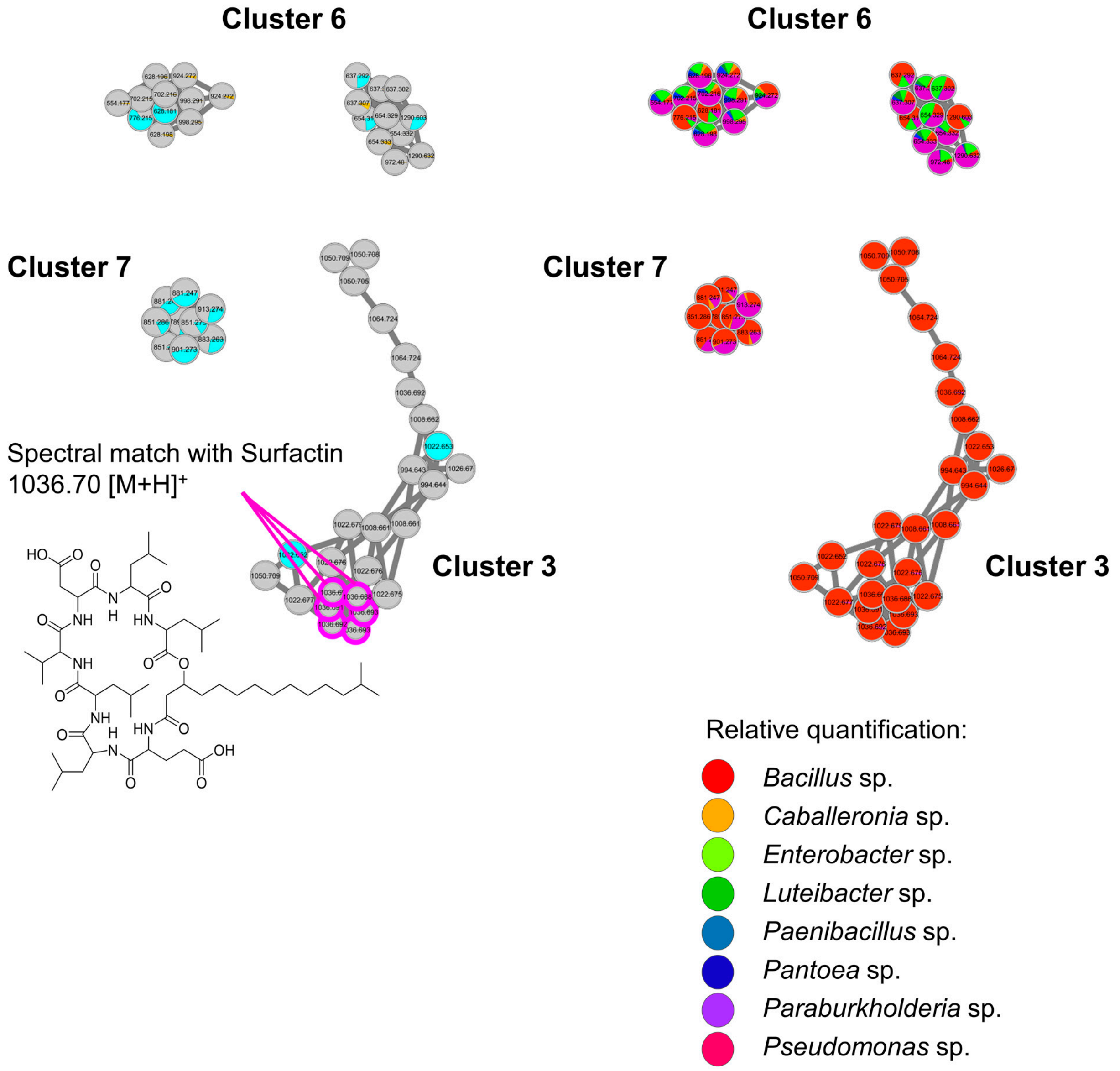

Figure S6. ^{1}H NMR spectrum of *(R)*-2-hydroxy-13-methyltetradecanoic acid **(1)** in CD_3OD

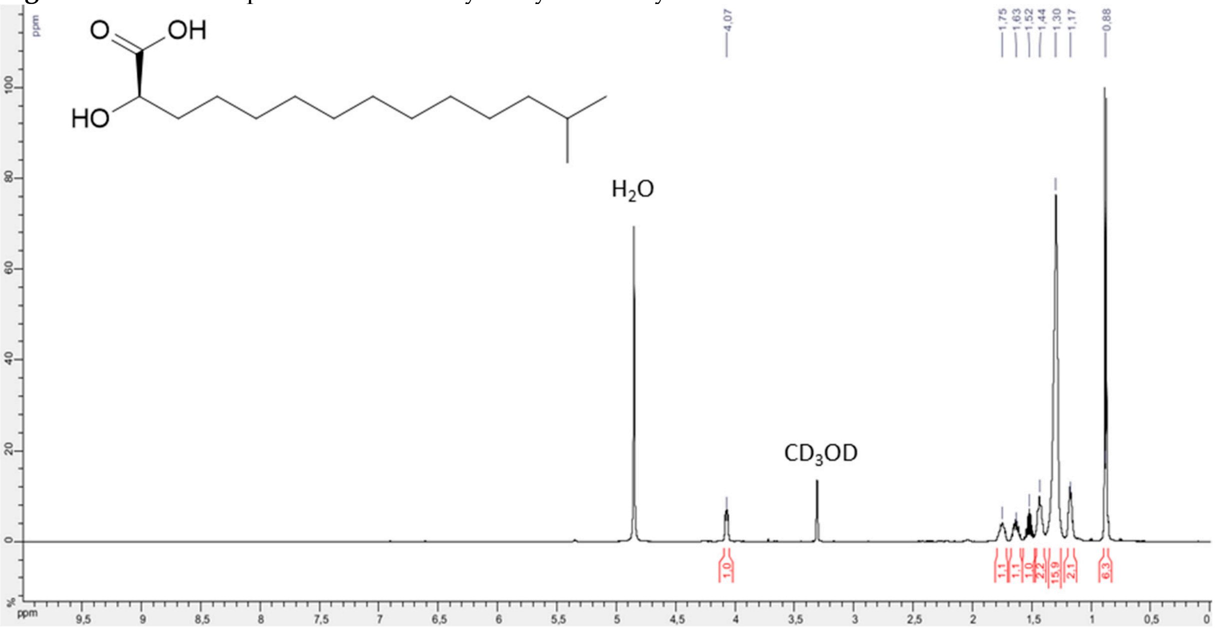

Figure S7. ^{13}C NMR spectrum of *(R)*-2-hydroxy-13-methyltetradecanoic acid **(1)** in CD_3OD

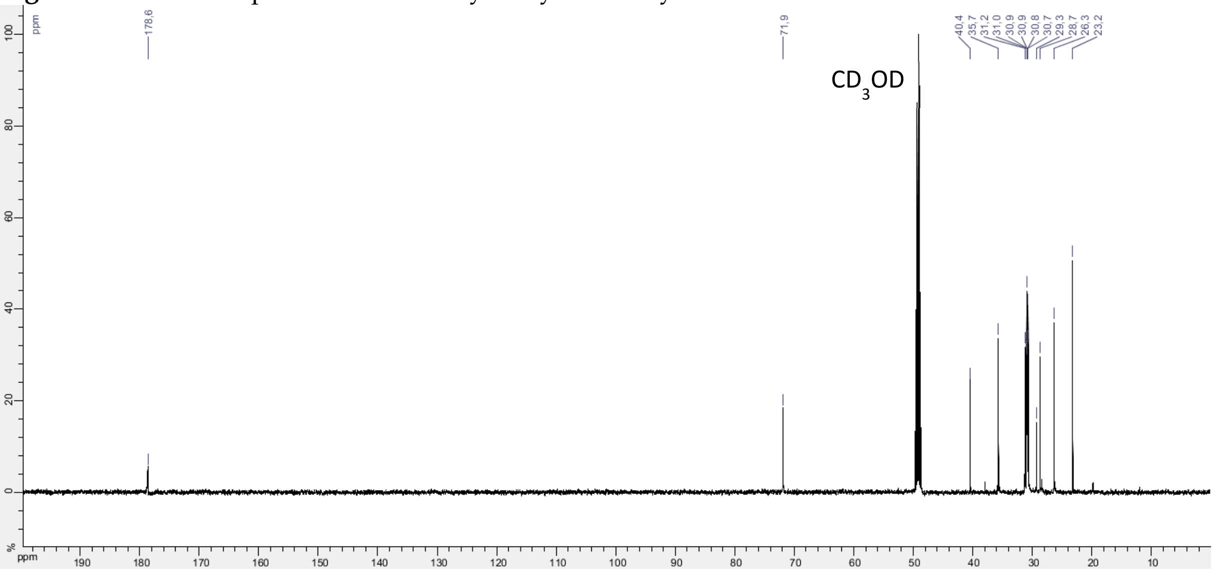

Figure S8. MS spectrum of *(R)*-2-hydroxy-13-methyltetradecanoic acid **(1)**

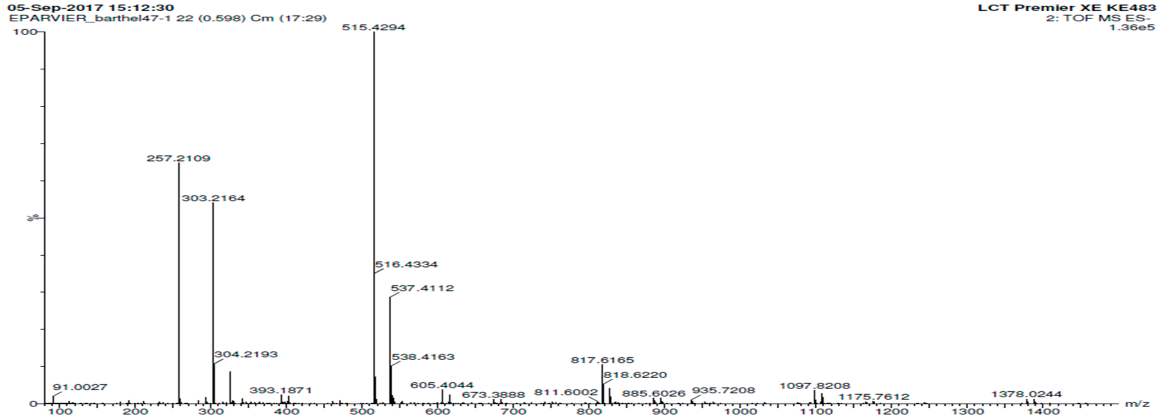

Figure S9. ^{1}H NMR spectrum of *(R)*-3-hydroxy-14-methyl-pentadecanoic acid **(2)**

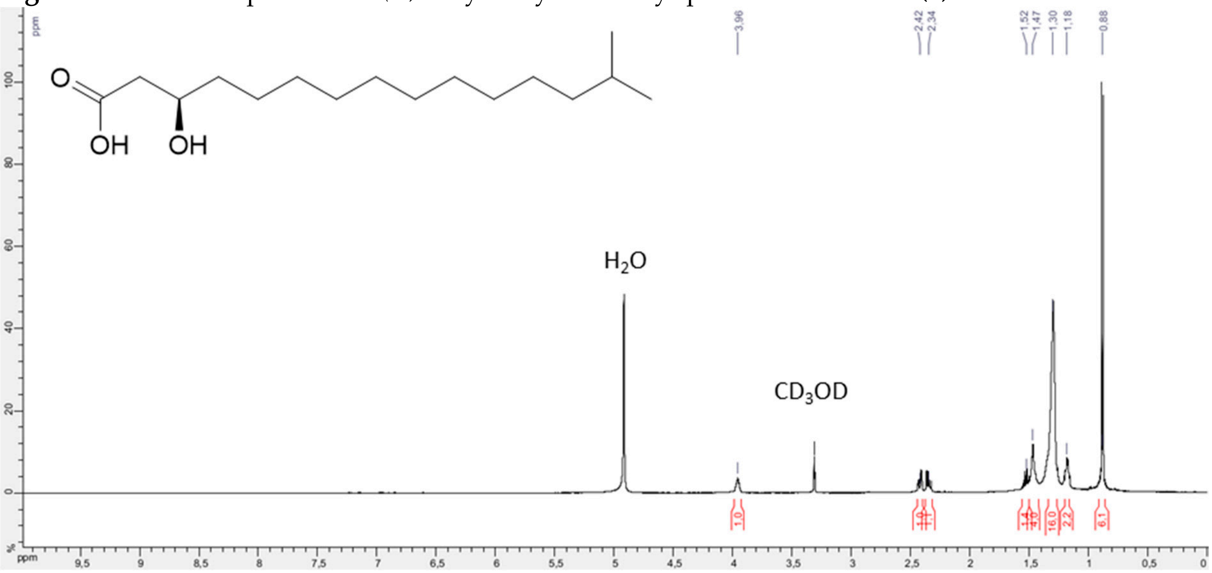

Figure S10. ^{13}C NMR spectrum of *(R)*-3-hydroxy-14-methyl-pentadecanoic acid **(2)**

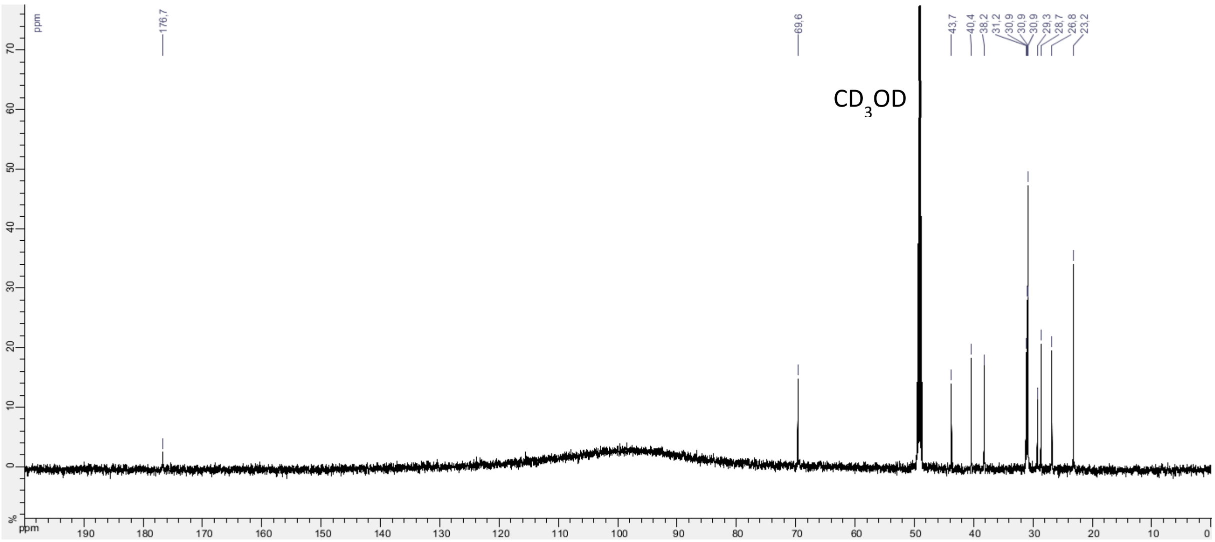

Figure S11. MS spectrum of *(R)*-3-hydroxy-14-methyl-pentadecanoic acid **(2)**

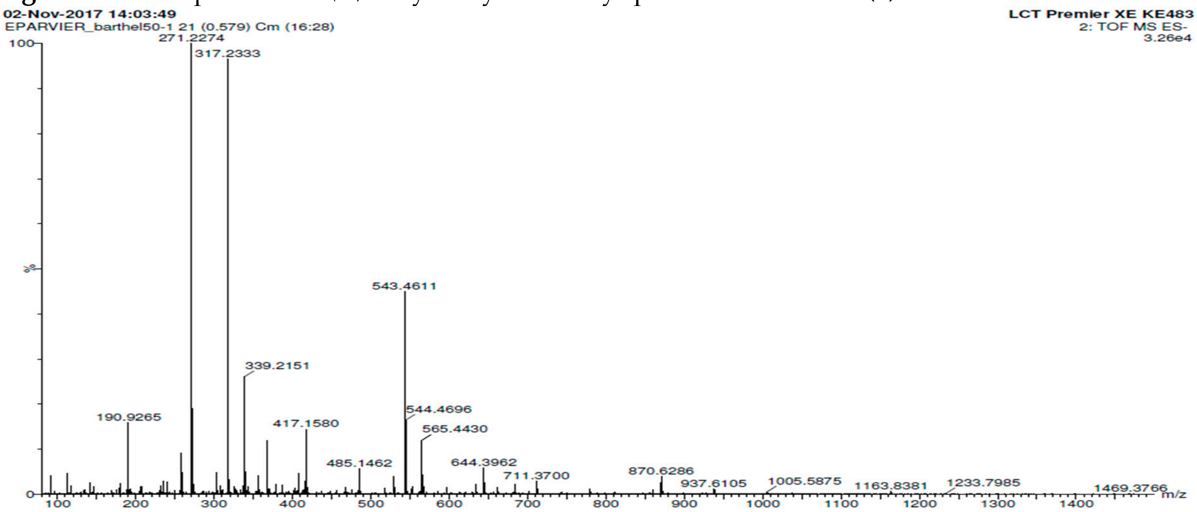

Figure S12. ^{1}H NMR spectrum of *(S)*-*β*-hydroxypalmitic acid **(3)**

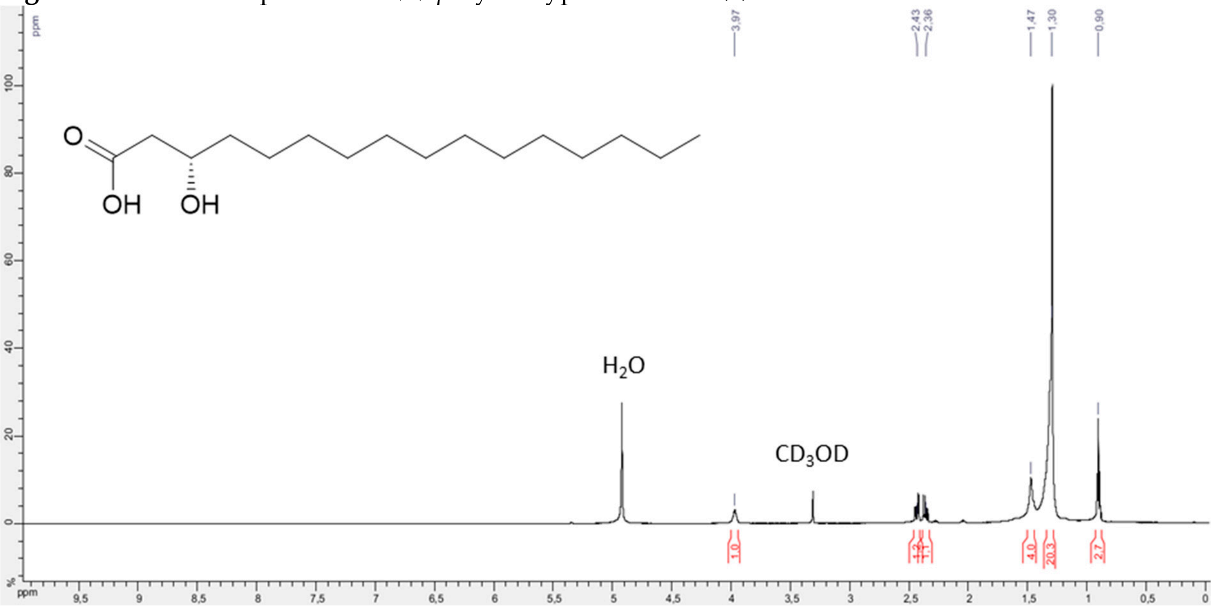

Figure S13. ^{13}C NMR spectrum of *(S)*-*β* -hydroxypalmitic acid **(3)**

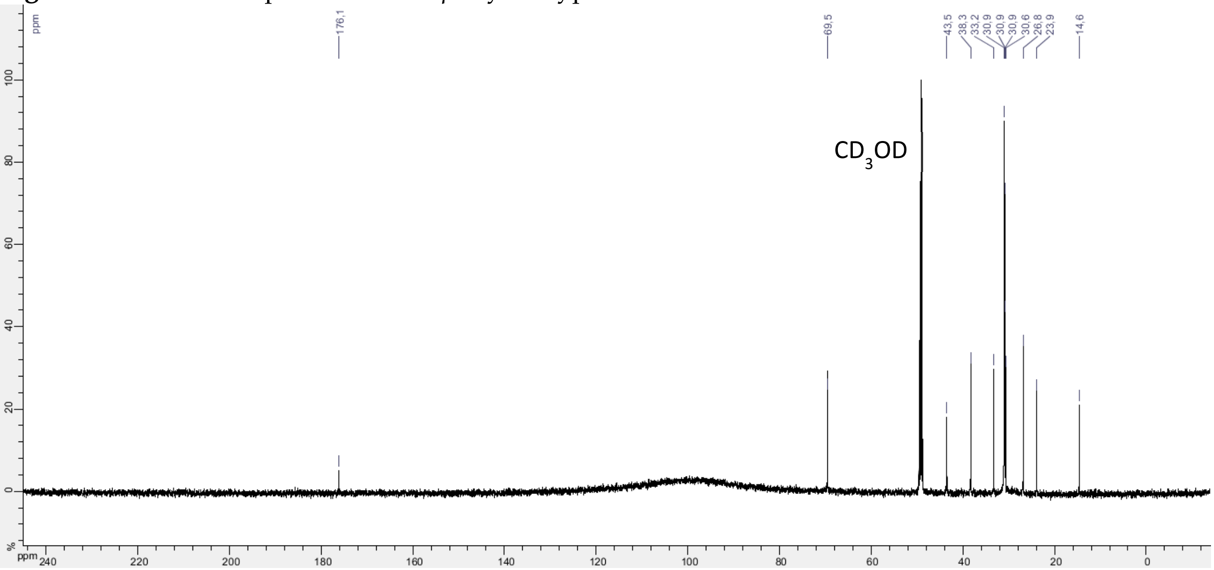

Figure S14. MS spectrum of *(S)*-*β*-hydroxypalmitic acid **(3)**

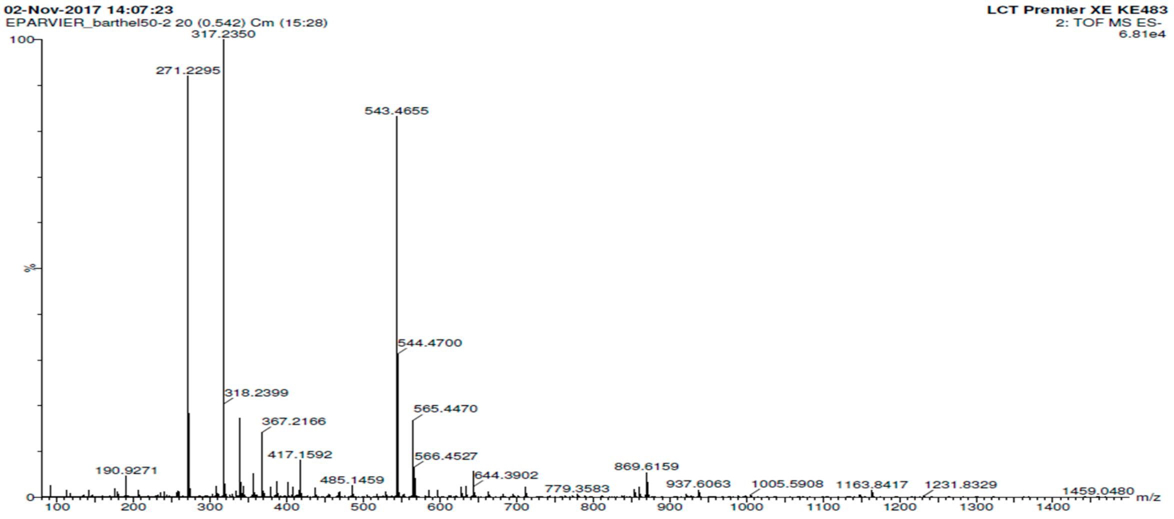

Figure S15. ^{1}H NMR spectrum of *(R)*-3-hydroxy-15-methylhexadecanoic acid **(4)** in CD_3OD

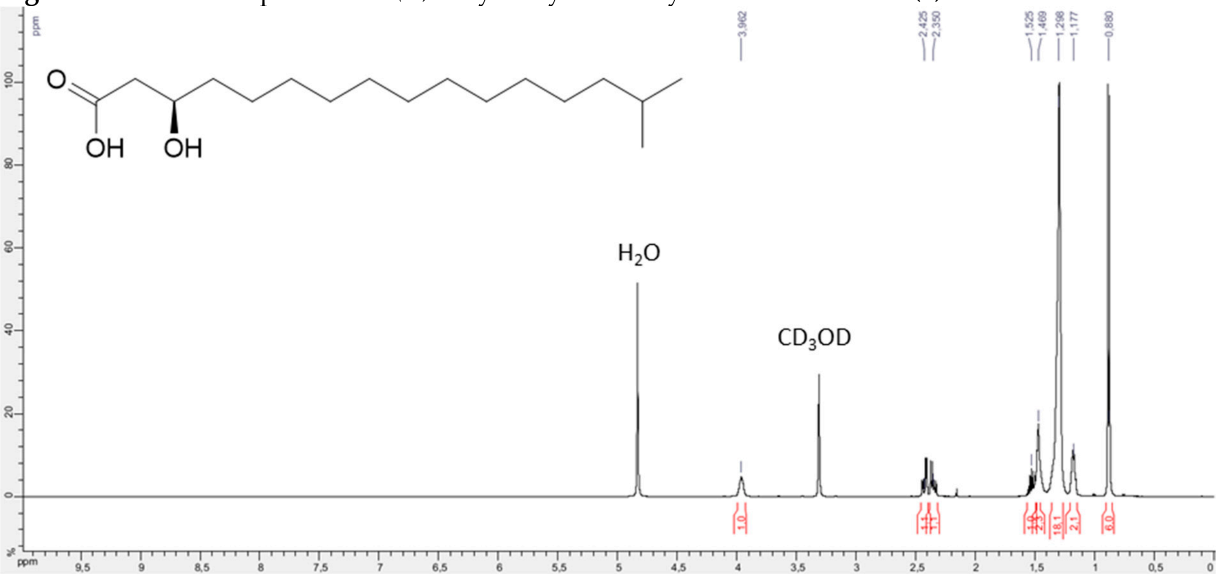

Figure S16. ^{13}C NMR spectrum of *(R)*-3-hydroxy-15-methylhexadecanoic acid **(4)** in CD_3OD

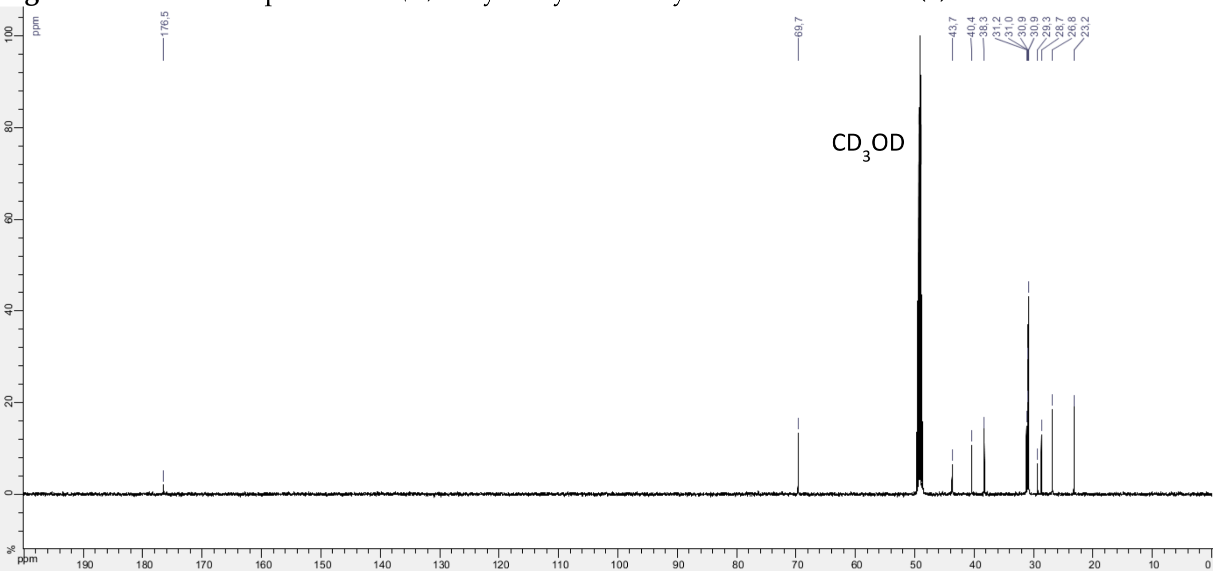

Figure S17. MS spectrum of *(R)*-3-hydroxy-15-methylhexadecanoic acid **(4)**

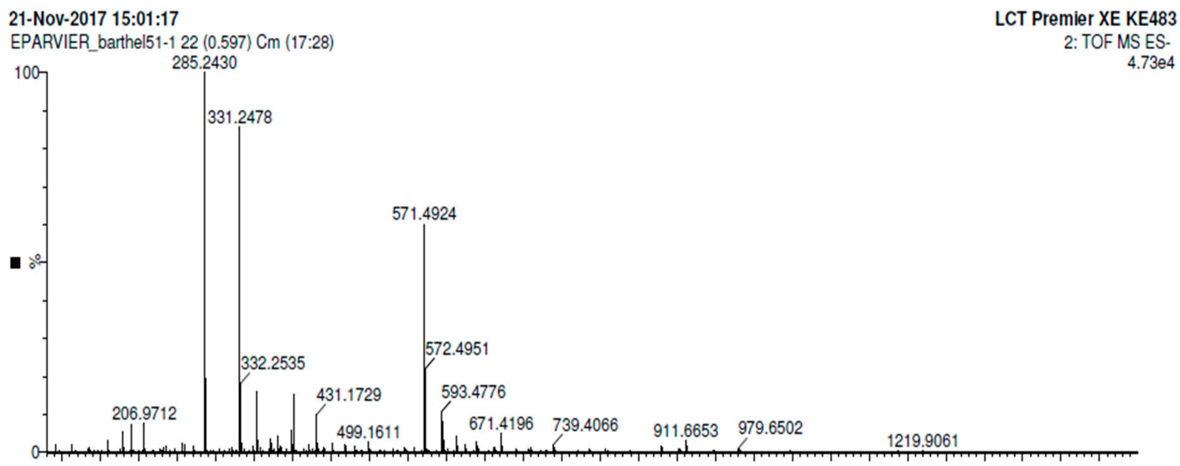

Figure S18. ^{1}H NMR spectrum of *(R)*-3-hydroxy-13-methyltetradecanoic acid **(5)** in CD_3OD

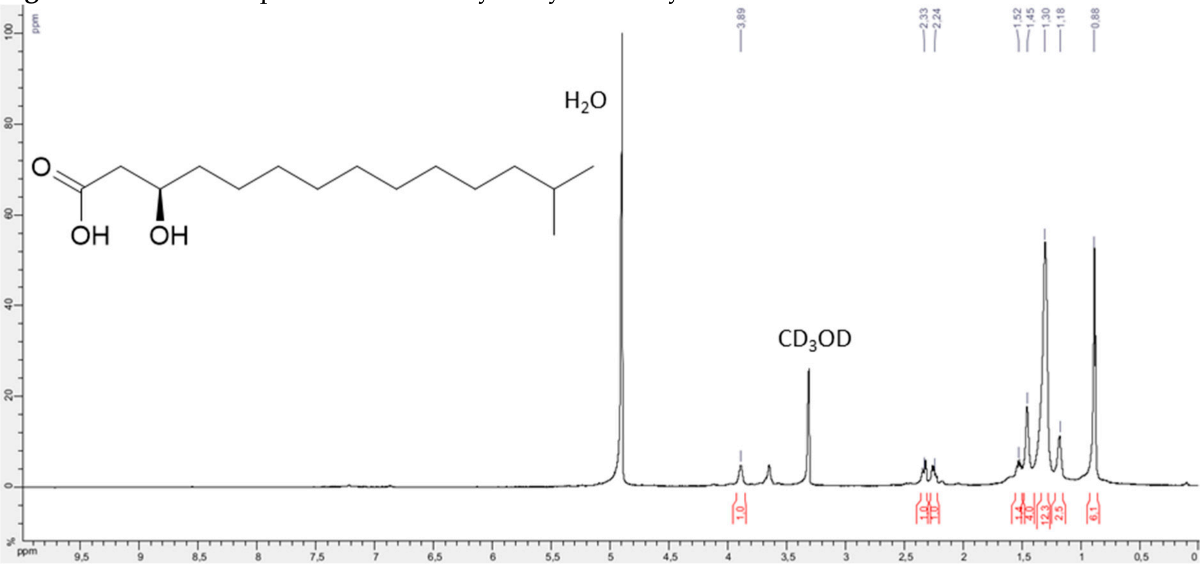

Figure S19. ^{13}C NMR spectrum of *(R)*-3-hydroxy-13-methyltetradecanoic acid **(5)** in CD_3OD

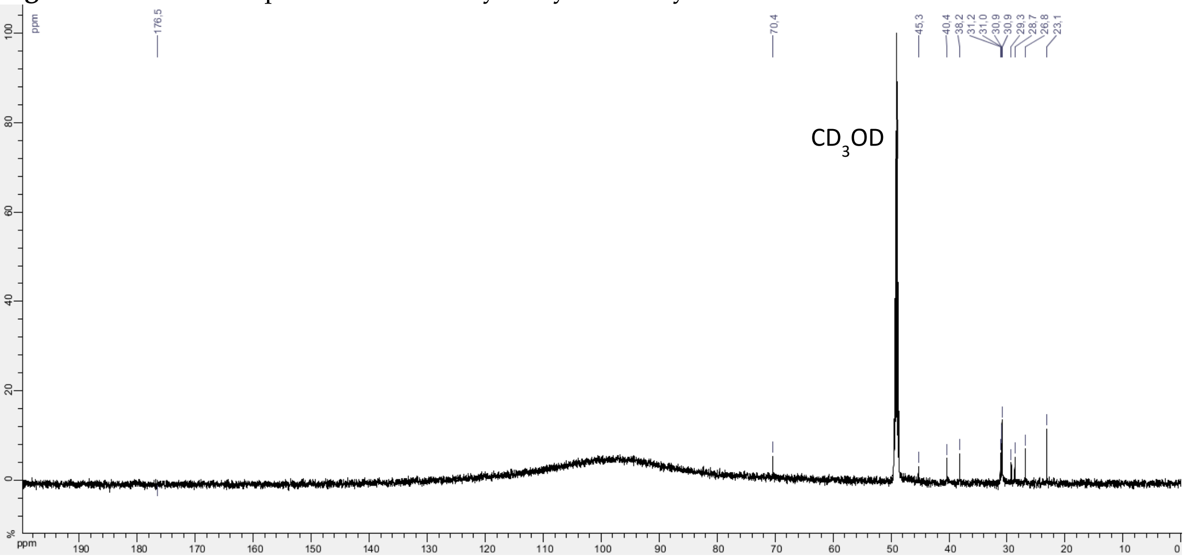

Figure S20. MS spectrum of *(R)*-3-hydroxy-13-methyltetradecanoic acid **(5)**

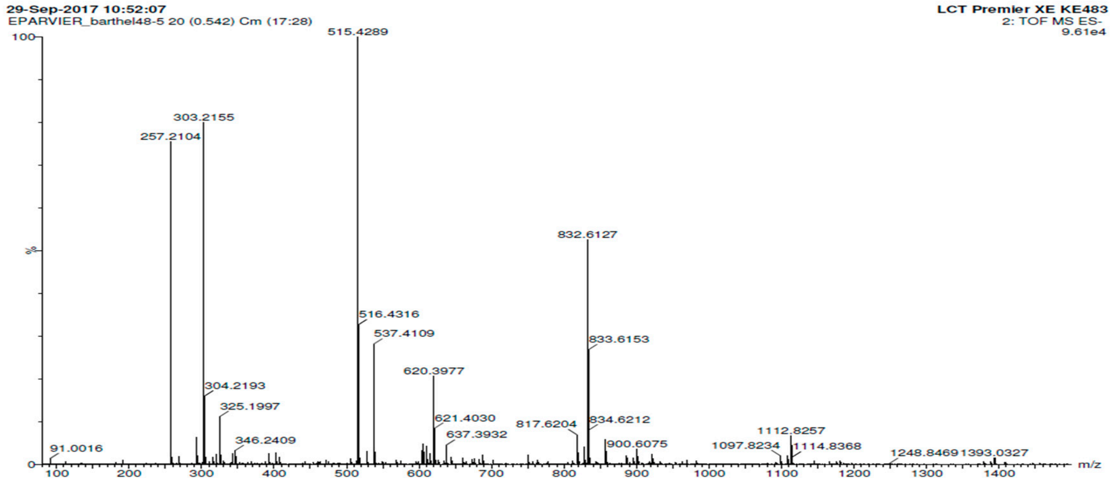

Figure S21. ^{1}H NMR spectrum of 13-methyltetradecanoic acid **(6)** in CD_3OD

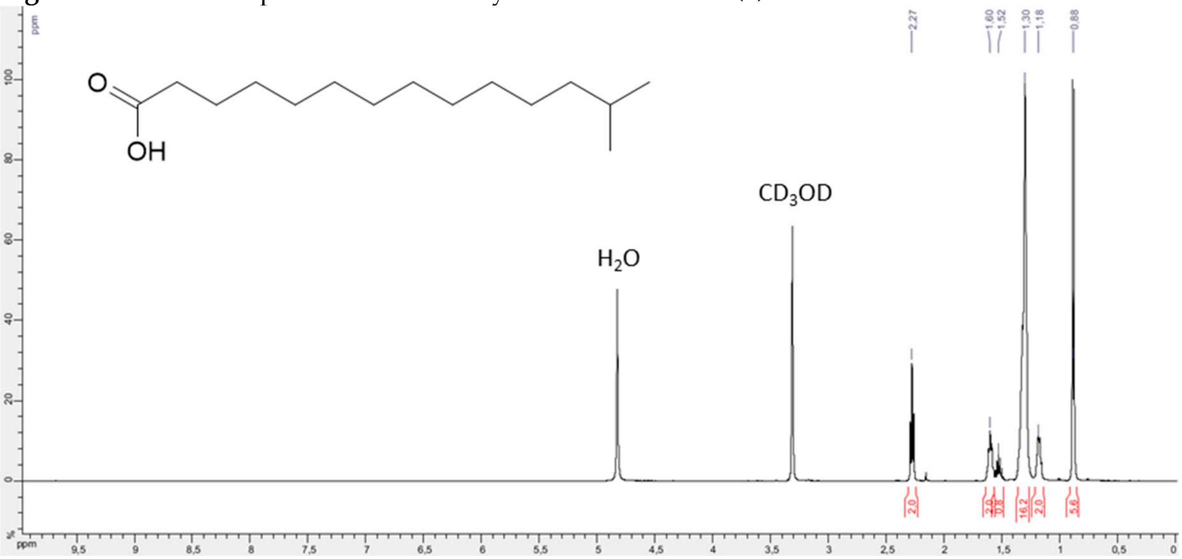

Figure S22. ^{13}C NMR spectrum of 13-methyltetradecanoic acid **(6)** in CD_3OD

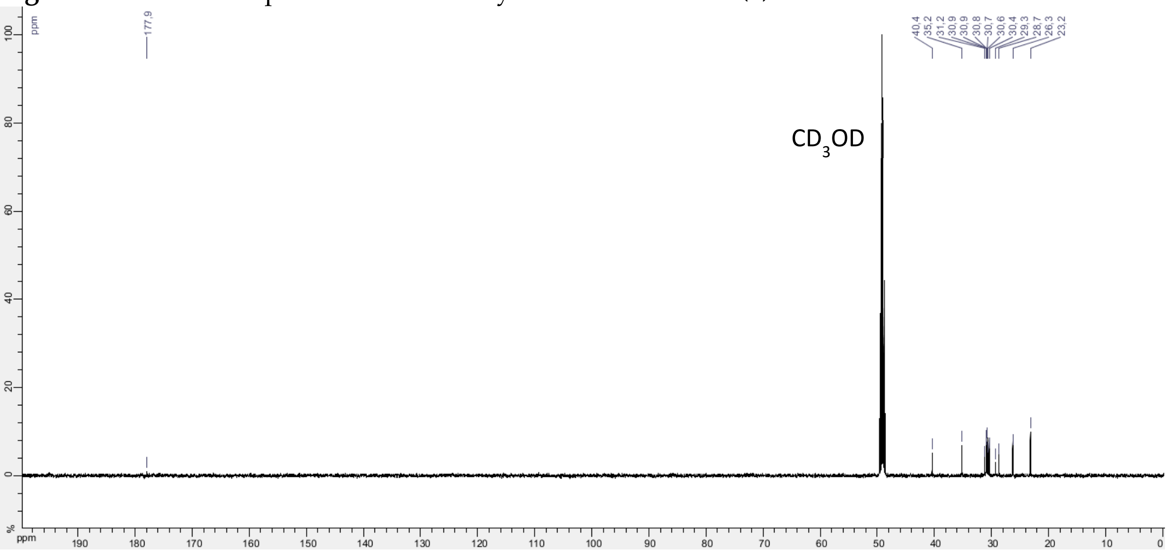

Figure S23. MS spectrum of 13-methyltetradecanoic acid **(6)**

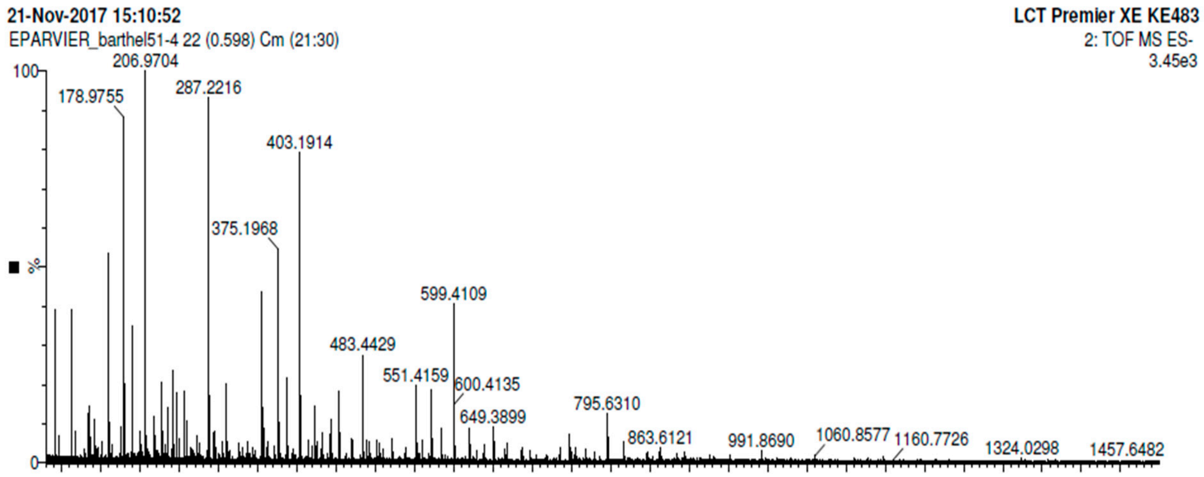

Figure S24. ^{1}H NMR spectrum of 9Z-hexadecenoic acid **(7)** in CD_3OD

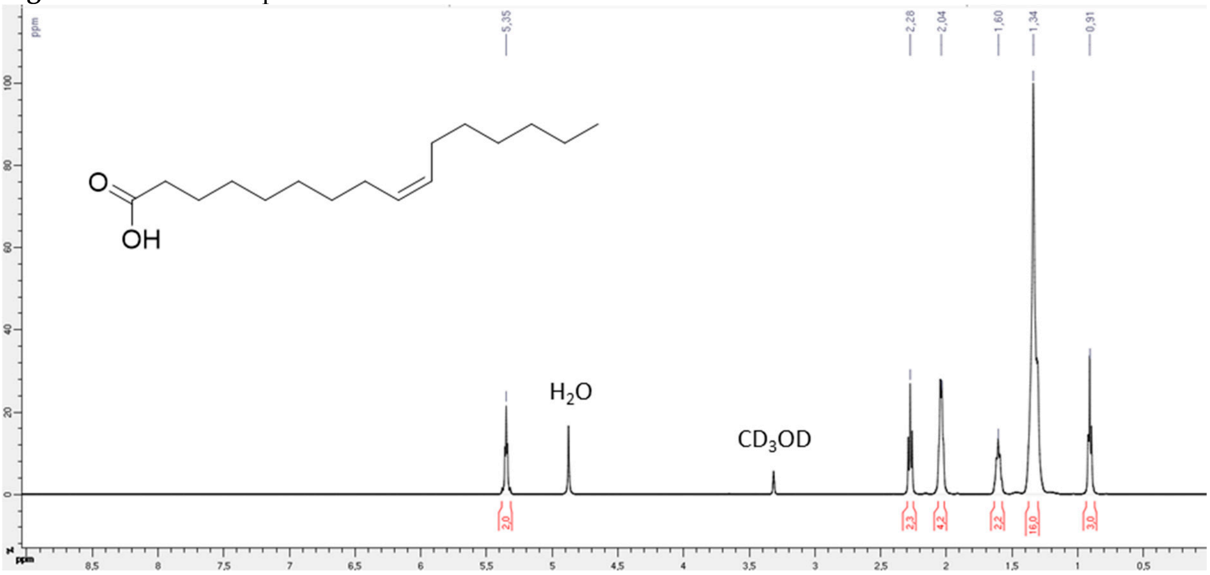

Figure S25. ^{13}C NMR spectrum of 9Z-hexadecenoic acid **(7)** in CD_3OD

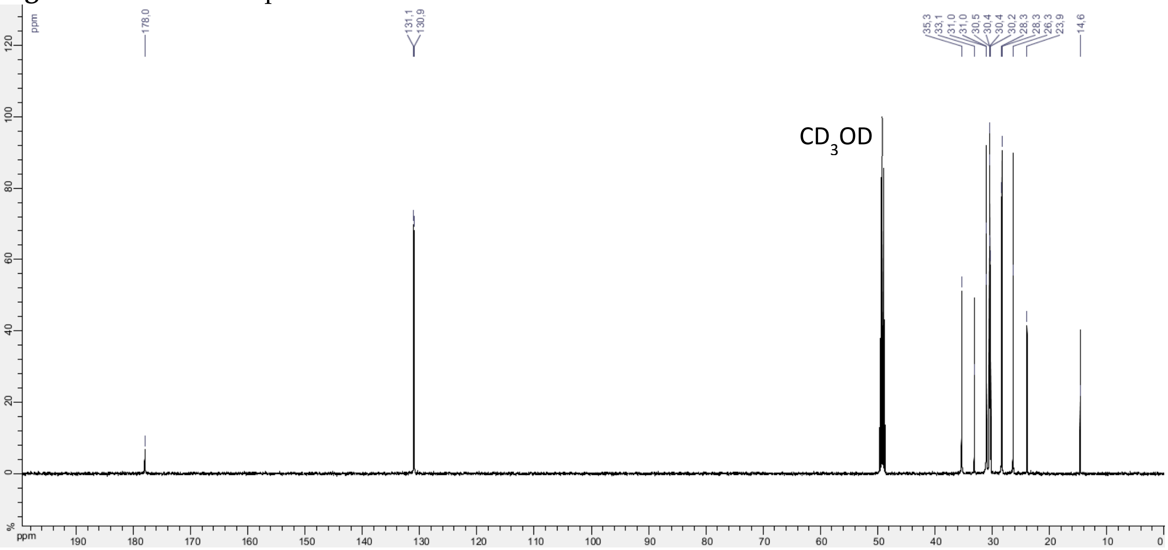

Figure S26. MS spectrum of 9Z-hexadecenoic acid **(7)**

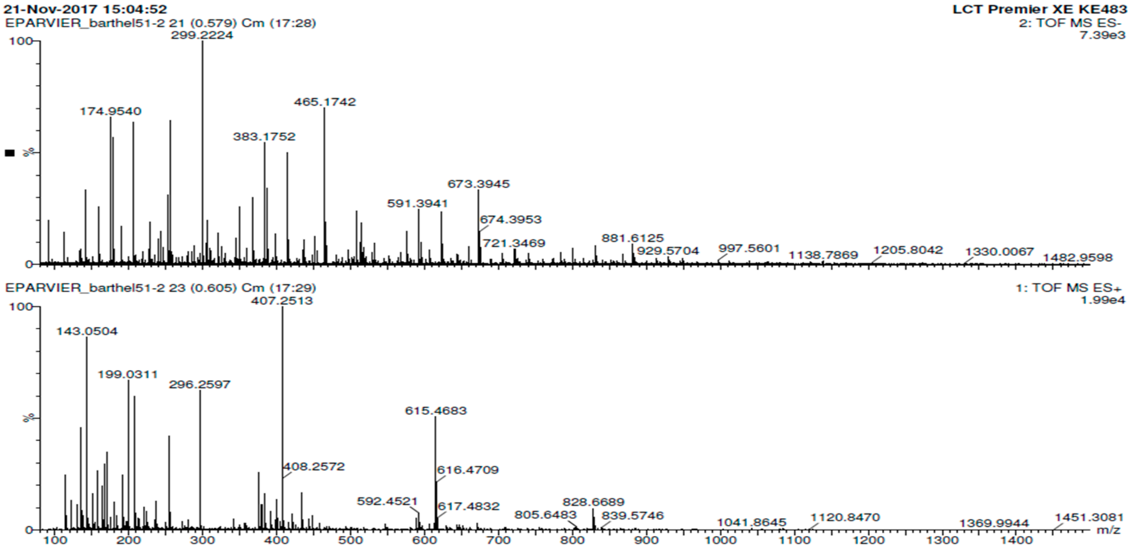

Figure S27. ^{1}H NMR spectrum of 15-methyl-9Z-hexadecenoic acid **(8)** in CD_3OD

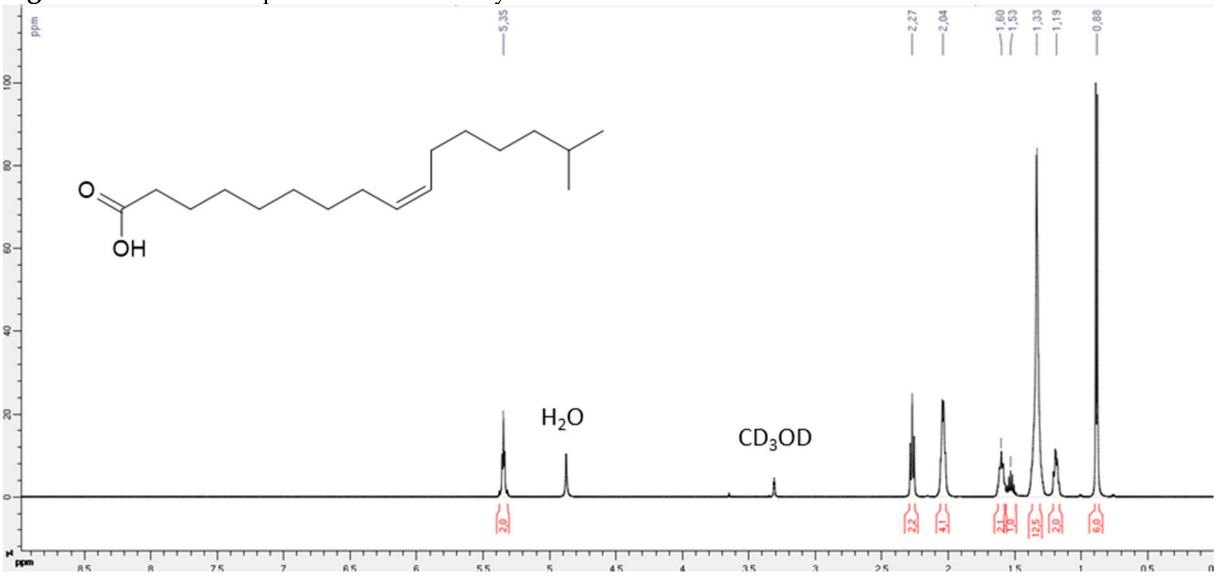

Figure S28. ^{13}C NMR spectrum of 15-methyl-9Z-hexadecenoic acid **(8)** in CD_3OD

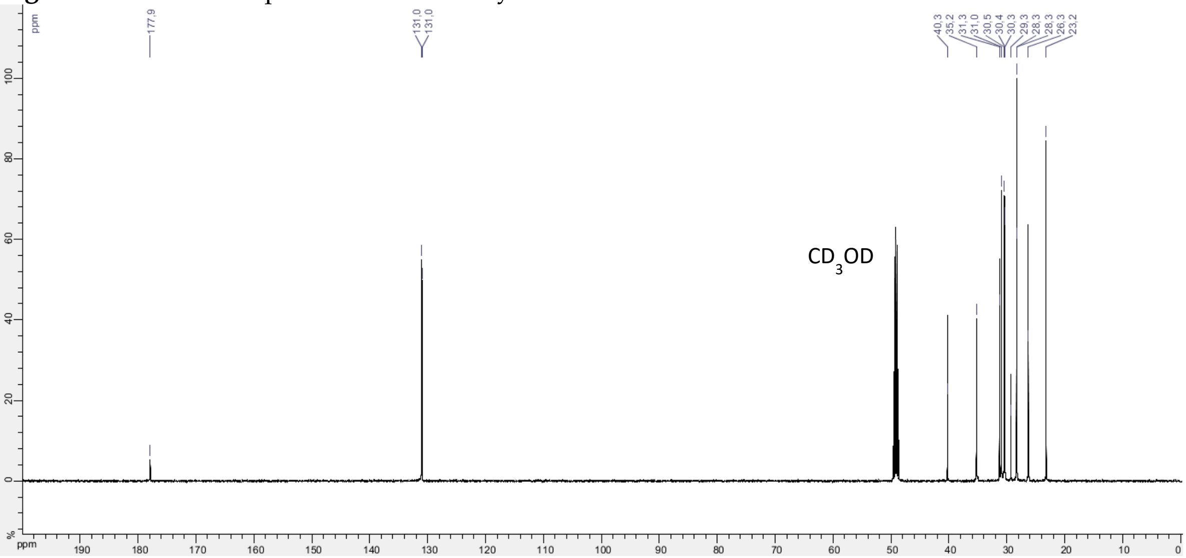

Figure S29. MS spectrum of 15-methyl-9Z-hexadecenoic acid **(8)**

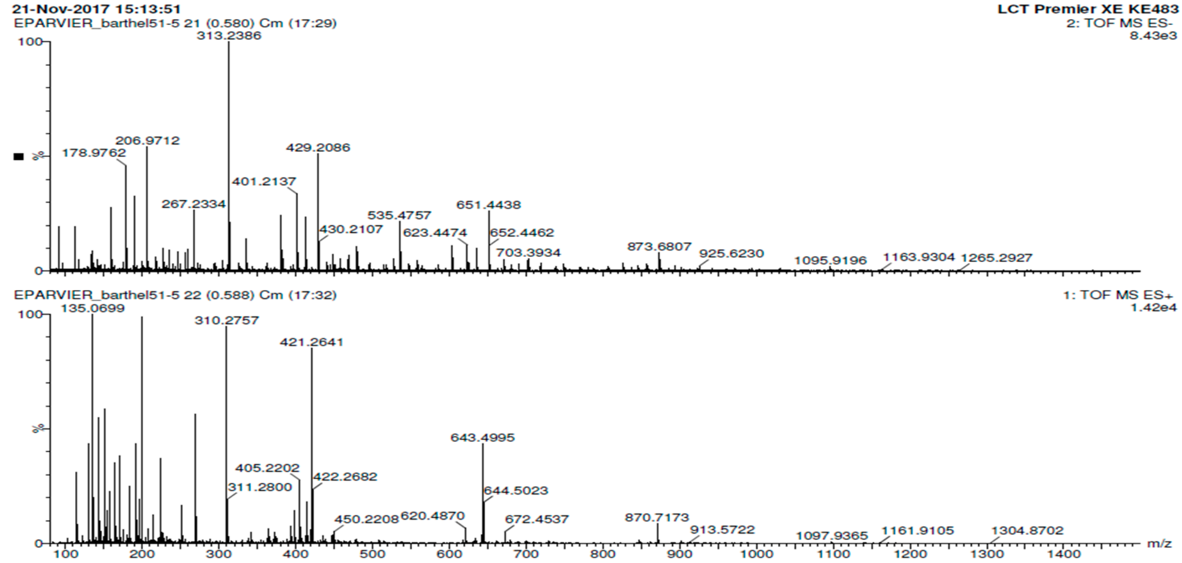

Figure S30. Fragmentation of 9Z-hexadecenoic acid **(7)**

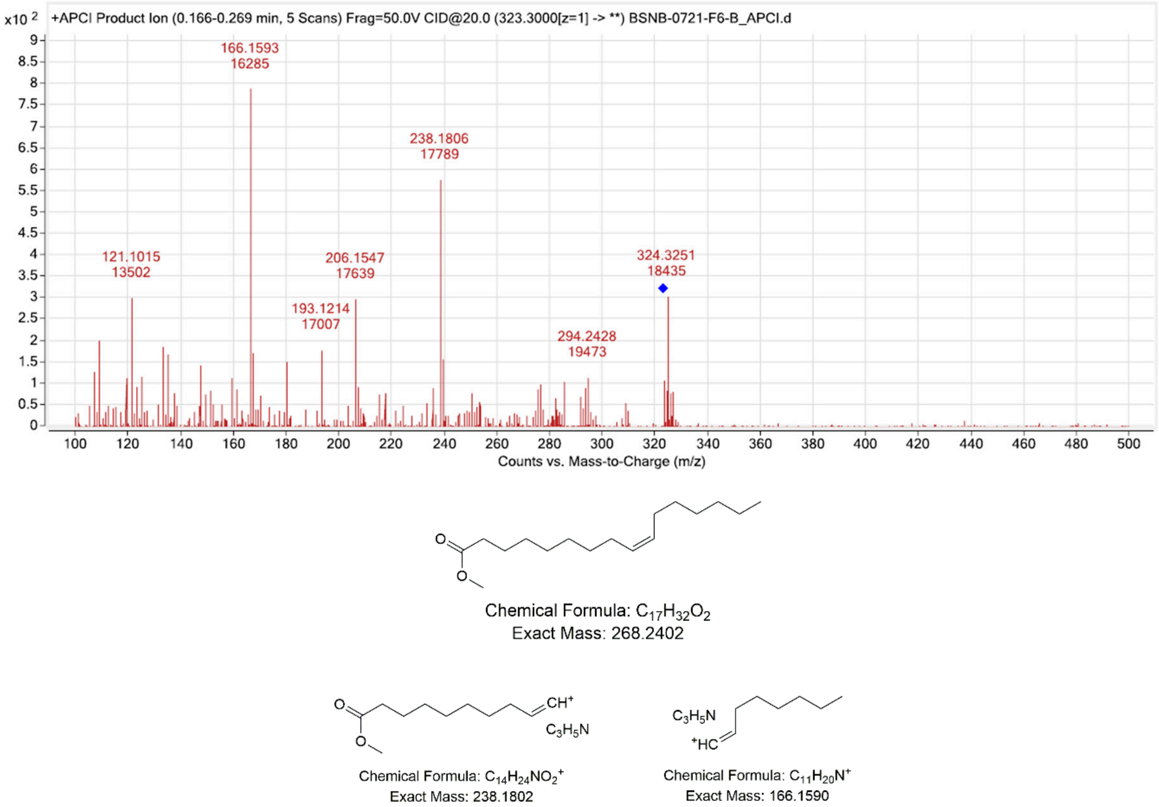

Figure S31. Fragmentation of 15-methyl-9Z-hexadecenoic acid **(8)**

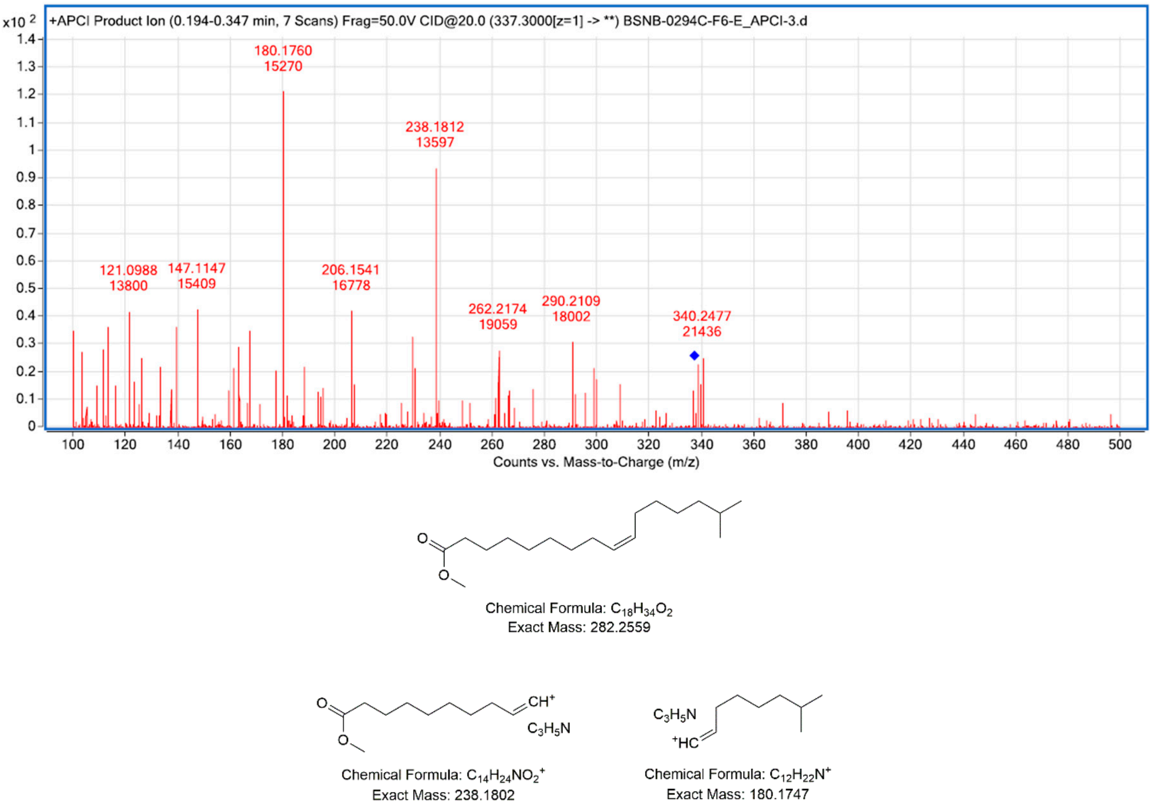

Table S1. Identification of endophytic microorganisms associated with *Astrocaryum sciophilum* and biological activities of their respective EtOAc extracts: antibacterial on methicillin-resistant *S. aureus* (MIC in µg/ml) and cytotoxicity on MRC-5 cells (viability percentage at the concentration of 10 µg/ml)

ID codes	Closest species in NCBI (accession number)	NCBI accession number	MRSA (µg/ml)	MRC-5	Selectivity index
BSNB-0575	*Fusarium* sp. (EF687915)	MK279517	32	20.2 ± 1.5	0.63
BSNB-0651	*Fusarium* sp. (EF687946)	MK279518	32	23.9 ± 3.0	0.75
BSNB-0303	*Fusarium decemcellular* (MH857667)	MK279519	16	14.8 ± 3.7	0.92
BSNB-0721	*Luteibacter* sp. (JQ723723)	MK279522	64	102.4 ± 4.5	1.60
BSNB-0730	*Bacillus subtilis* (CP029461)	MK279521	64	106.5 ± 3.5	1.66
BSNB-0732	*Akanthomyces attenuatus* (MH872738)	MK279520	< 8	5.0 ± 0.1	> 0.62

Positive Control: Vancomycin against MRSA (MIC = 1 µg/ml) and Docetaxel against MRC-5 cells (IC_{50} = 5×10^{-10} M, 0 % viability at 10 µg/ml)

Table S2. Antibacterial activity of AcOEt extract of *Luteibacter* sp. and its fractions on MRSA (Minimum Inhibitory Concentration in µg/mL)

ID codes	Fractions	MRSA (µg/mL)
BSNB-0721	Crude extract	64
	F1	> 256
	F2	> 256
	F3	> 256
	F4	128
	F5	256
	F6	32
	F7	256
	F8	> 256

Positive control: Vancomycin (MIC = 1 µg/mL)
